# Monofloral Honeys as a Potential Source of Natural Antioxidants, Minerals and Medicine

**DOI:** 10.3390/antiox10071023

**Published:** 2021-06-25

**Authors:** Rodica Mărgăoan, Erkan Topal, Ralitsa Balkanska, Banu Yücel, Titanilla Oravecz, Mihaiela Cornea-Cipcigan, Dan Cristian Vodnar

**Affiliations:** 1 Advanced Horticultural Research Institute of Transylvania, University of Agricultural Sciences and Veterinary Medicine Cluj-Napoca, 400372 Cluj-Napoca, Romania; 2 Apiculture Research Center, Aegean Agricultural Research Institute, İzmir 35100, Turkey; topalerkan@tarimorman.gov.tr; 3 Department of Special Branches-Bees, Institute of Animal Science, Spirka Pochivka 1, 2232 Kostinbrod, Bulgaria; obi4liv4e@mail.bg; 4 Department of Animal Science, Faculty of Agriculture, Ege University, İzmir 35100, Turkey; banu.yucel@ege.edu.tr; 5 Marketing Department, Faculty of International Management and Business, Budapest Business School, Diósy Lajos u. 22-24., H-1165 Budapest, Hungary; oravecztitanilla@gmail.com; 6 Faculty of Horticulture, University of Agricultural Sciences and Veterinary Medicine Cluj-Napoca, 400372 Cluj-Napoca, Romania; 7 Department of Food Science, University of Agricultural Sciences and Veterinary Medicine Cluj-Napoca, 400372 Cluj-Napoca, Romania; dan.vodnar@usamvcluj.ro or

**Keywords:** monofloral honey, phenolic compounds, antioxidant activity, physicochemical properties, anticancer

## Abstract

Background: vegetative diversity is based on different climate and geographical origins. In terms of beekeeping, herbal diversity is strongly correlated to the production of a wide variety of honey. Therefore, based on the existing plant diversity in each country, multiple honey varieties are produced with different health characteristics. While beekeeping potential and consumption preferences are reflected in products’ variety, this leads to an increase in the region’s economy and extensive export. In the last years, monofloral honey has gained interest from consumers and especially in the medicinal field due to the presence of phytochemicals which are directly linked to health benefits, wound healing, antioxidant, anticancer and anti-inflammatory activities. Scope and approach: this review aims to highlight the physicochemical properties, mineral profiles and antioxidant activities of selected monofloral honeys based on their botanical and geographical origin. Moreover, this review focuses on the intercorrelation between monofloral honey’s antioxidant compounds and in vitro and in vivo activities, focusing on the apoptosis and cell proliferation inhibition in various cell lines, with a final usage of honey as a potential therapeutic product in the fight towards reducing tumor growth. Key findings and conclusions: multiple studies have demonstrated that monofloral honeys have different physicochemical structures and bioactive compounds. Useful chemical markers to distinguish between monofloral honeys were evidenced, such as: 2-methoxybenzoic acid and trimethoxybenzoic acid are distinctive to Manuka honey while 4-methoxyphenylacetic acid is characteristic to Kanuka honey. Furthermore, resveratrol, epigallocatechin and pinostrobin are markers distinct to Sage honey, whereas carvacrol and thymol are found in Ziziphus honey. Due to their polyphenolic profile, monofloral honeys have significant antioxidant activity, as well as antidiabetic, antimicrobial and anticancer activities. It was demonstrated that Pine honey decreased the MDA and TBARS levels in liver, kidney, heart and brain tissues, whereas Malicia honey reduced the low-density lipoprotein level. Consumption of Clover, Acacia and Gelam honeys reduced the weight and adiposity, as well as trygliceride levels. Furthermore, the antiproliferative effect of chrysin, a natural flavone in Acacia honey, was demonstrated in human (A375) and murine (B16-F1) melanoma cell lines, whereas caffeic acid, a phenolic compound found in Kelulut honey, proves to be significant candidate in the chemoprevention of colon cancer. Based on these features, the use of hiney in the medicinal field (apitherapy), and the widespread usage of natural product consumption, is gaining interest by each year.

## 1. Introduction

Honey bees (*Apis mellifera* L.) are the main pollinators in the world, providing the highest service in crop pollination. They pollinate 90% of the globally most important crops [1]. Managed honey bees ensure a billion euros in crop production worldwide each year [2]. In some countries, such as the USA (United States of America), pollination service is the primary source of income for beekeepers, who use it to pollinate almond orchards [3,4]. There is clear evidence of recent declines in both wild and domesticated pollinators and parallel declines in plants that depend on them [5]. Pollinator declines can result in the loss of pollination services, which has ecological and economic impacts which can significantly affect wild plant diversity, ecosystem stability, crop production, food security and human well-being [5,6]. In the last decade, high levels of bee losses have been reported all over the world. Many factors contribute to the dramatic bee colony losses every year. The most important ones are as follows: agrochemical exposure; the use of fungicides, herbicides and insecticides, “chemisation” in agriculture; bee diseases, nutritional stress, environmental pollution from toxic metals, nitrogen and light, as well as global climate changes [7,8,9,10,11]. Indeed, very often colony losses are the result of the effect of different and complicated factors.

Apart from pollination, many bee products are obtained from honey bees. Honey is the most highly produced bee product in the world. Additionally, products such as bee pollen, bee bread, royal jelly, apilarnil, queen bee larvae, propolis and the bee itself are consumed as food in many countries [12,13,14,15].

Honey is a natural sweetening food item which has been consumed by humans for thousands of years. It consists of many macro and micro components such as carbohydrates, water, enzymes, proteins, vitamins, organic and amino acids, phenolic compounds, pollen particles, essential oils and sterols. The physical and chemical composition of honey varies according to its botanical origin, the region where it is produced and the processes applied. In addition to its high nutritional value, it has been found that honey has antimicrobial, antioxidant, anti-inflammatory, antimutagenic and anticarcinogenic effects [16,17,18,19,20]. The main compounds responsible for the antioxidant activity of honey are flavonoids (chrysin, pinosembrine, quercetin, galangin, kaempferol, hesperetin and myceticine), phenolic acids (caffeic, coumaric, ellagic, ferulic and chlorogenic acids), ascorbic acid, catalase, peroxidase, carotenoids and maillards. Honey’s antibacterial effect is due to its high osmolarity, low pH, hydrogen peroxide, glucose oxidase enzyme, honeybees’ hypopharyngeal secretions, catalase activity resulting from flower pollen and nectar, propolis and its phenolic derivatives [21].

Considering the distinct physicochemical composition and phenolic compounds found in monofloral honeys, this review aimed to highlight the antioxidant, as well as the in vitro and in vivo, activities, focusing on the apoptosis and cell proliferation inhibition in various cell lines, with a final usage of monofloral honeys as a potential therapeutic product in the fight towards reducing tumor growth.

In this review, firstly, the herbal diversity of monofloral honeys is introduced, followed by their antioxidant activity and potential medicinal properties. Furthermore, their physicochemical properties and mineral composition are investigated.

## 2. Methods of Review

A literature search from 1999 to present in Google Scholar, PubMed and ScienceDirect was conducted to identify and select relevant and significant articles related to the mineral, phenolic compounds and medicinal properties of monofloral honeys. 

Keywords including monofloral, unifloral, honey, minerals, phenolics, flavonoids, in vitro, in vivo, anticancer, cell lines and the names of several countries were applied. Natural honey or honeys with unknown species were excluded as they do not meet the purpose of the present study.

## 3. Herbal Diversity of Monofloral Honeys

Vegetative diversity increases because of country sizes, soil types and climatic diversity. Even though monofloral honeys are produced at a global level, there are cases in which these specific honeys are solely produced for their representative countries.

The honeys available in the market differ in quality due to various factors such as geographical, seasonal and processing conditions, as well as flower source and packaging and storage conditions. While the sensory property of honey represents an important parameter in determining its quality for consumers, the biggest effect is related to the colour and crystallization state [22]. Floral variety allows the production of a wide range of economically important honeys (Table 1).

Scientific studies revealed the many properties of compounds isolated from honey. With these features, honeys offer multi-purpose usage possibilities. Monofloral honey is obtained from the nectar of specific source plants and may prove to be more valuable than polyfloral honey. Therefore, the type of honey should be determined before labelling and marketing [59,60,61].

Various types of honeys are produced in Turkey, mostly Chestnut, Pine, Rhododendron, Milkvetch and Thyme. Bulgarian and Hungarian honeys are more complex, predominantly composed of the following species: Coriander, Thistle, Buckwheat, Willow and Linden, as well as forest honeys. Romanian and Ukrainian monofloral honeys are represented by Canola, Chestnut, Linden, Sunflower and Willow. The honeys from Portugal are rather diverse, being predominant in Buckthorn, Cytisus, Citrus, Eucalyptus, Heather, Lavender, Portugal laurel and Thyme. There are various types of eucalyptus honeys with different chemical constituents based on the geographical origin or production region. Recently, it has been demonstrated that eucalyptus honey has a significant antioxidant activity and it can show antimicrobial effects against many microorganisms [61]. As stated by the National Institute of Standardisation UNI, there are five main Italian monofloral honeys, as follows: Black Locust, Chestnut, Citrus, Eucalyptus and honeydew honey. Aside from these, there are several specific honeys, such as: Asphodel, Eucalyptus, Linden, Clover, Marruca (*Paliurus spina-christi*), Sulla and Sunflower.

The honeys from Malaysia and Australia are mainly Tualang and Gelam honeys, whereas from New Zealand are Clover, Manuka and Thyme honeys. Manuka honey is collected from *Leptospermum scoparium* or *Leptospermum polygalifolium*, of the Myrtaceae family, which can be found throughout New Zealand and eastern Australia [55,56]. Kanuka honey is derived from *Kunzea ericoides*, as a member of the same botanical family as the manuka tree and, according to Semprini et al. (2019) [57], kanuka-derived honey may offer similar antiviral effects. Tualang honey is rich in phenolic acids, flavonoids and has significant anticancer activity. It is collected from *Koompassia excels*, which is found in the Malaysian rain forest [53,54].

## 4. Phenolic Compounds

The antioxidant activity altogether with the in vitro activities of honey from various floral sources emphasizes the importance of integrating monofloral honey as a daily consumption product. Multiple reports highlight that rich phenolic compounds and the antioxidant capacity of monofloral honeys play a significant role in the expression of a wide range of bioactivities. The most common phenolic compounds in monofloral honeys are shown in Table 2.

In Acacia honeys, eight phenolic compounds and abscisic acid were identified in almost all samples and seemed to exhibit higher levels. These characteristic compounds included vanillic acid, caffeic acid, *p*-coumaric acid, ferulic acid, 3,4-dimethoxycinnamic acid, cinnamic acid, abscisic acid, kaempferol and pinocembrin. These nine characteristic compounds represent practically 50% of the total phenolics. In Serbian honeys, Kečkeš et al., (2013) [72] demonstrated that Sunflower, Basil, Buckwheat, Canola and Goldenrod honeys are strongly correlated to the phenolic profile. Thus, they demonstrated that quercetin and eriodictyol could be used as floral markers for Serbian honey, as well as *cis*, *trans*-Abscisic acid, which clearly distinquished between acacia and linden honeys.

The chemical constituents of Peppermint honey revealed that the most common compounds are *p*-coumaric acid and kaempferol, as well as a high abundance of methyl syringate, vomifoliol, 3,7-dimethylocta-1,5-dien-3,7-diol (terpendiol I) and hotrienol. Furthemore, the major honey headspace compounds were hotrienol, *cis*- and *trans*-linalool oxides, linalool and neroloxide [86].

Useful chemical markers such as carvacrol and thymol were solely found in *Ziziphus Spina-Christi* honey, as well as kojic acid distinctive to *Coffea* sp. honey [92,93], Quercetin 3′,3′-dimethyl eter and Quercetin 7,3′-dimethyl eter characteristic in Rosemary honey (Arráez-Román et al., 2006) [69] and resveratrol distinctive to Sage (*Salvia officinalis* L.) honey (Gašić et al., 2015) [65]. Furthermore, several compounds are useful to discriminate honeys with similar properties and palinological characteristics. This is the case of Manuka (*Leptospermum scoparium*) and Kanuka (*Kunzea erikoides*) honeys, which are not distinguished by means of a melissopalynological analysis. Even though they share most of their phenolic profiles, Stephens et al., 2010 [89] noticed that 2-methoxybenzoic acid and trimethoxybenzoic acid are distinctive to Manuka honey, while 4-methoxyphenylacetic acid is characteristic to Kanuka honey. Furthermore, an increasing interest has been noticed in using the polyphenolic profile to distinguish honeys based on their geographic origin. Habib et al., 2014 [79] showed dissimilarities in the phenolic pattern based on the different climate and sunlight exposure. According to their study, sunlight exposure is responsible for higher polyphenol content in honeys produced in dry areas. The phenolic profile of Serbian unifloral honeys was investigated by Kečkeš et al., 2013 [72], who proposed eriodictyol and quercetin as floral markers for local Sunflower honeys. Furthermore, the phenolic profile of Sulla honeys produced in Southern Italy were influenced by their geographical origin [94], whereas the concentrations of gallic, chlorogenic, caffeic, *p*-coumaric and ferulic acids showed the highest variation as a function of the production site of this honey. Furthermore, Weston et al. (1999) [95] presented several methods for isolation of the antibacterial active phenolic fraction of honey derived from the native New Zealand manuka tree *Leptospermum scoparium*. This fraction consists of phenolic derivatives of benzoic acids, cinnamic acids and flavonoids, all of which have been identified previously in honeys. Regarding manuka honeys, the major flavonoids are pinobanksin, pinocembrin and chrysin, whereas galagin, isorhamnetin, kaempferol, 8-methoxykaempferol, luteolin and quercetinare were identified in minor concentration [96,97].

## 5. Physicochemical Properties of Monofloral Honeys

Honey, a high-quality natural product with a complex chemical composition, contains a wide range of nutritional and therapeutic properties.

The quality is usually assessed by physicochemical, sensorial and microbiological parameters. The physicochemical properties depend on the type of nectar (botanical origin), geographical origin (climate conditions and soil composition) and handling (storage and transport). Multiple studies have reported that the physicochemical parameters of honey, such as moisture, acidity, sugar composition, ash and 5-hydroxymethylfurfural (HMF) are used to distinguish honey types. The physicochemical characteristics of monofloral honeys from different countries are exemplified in Table 3.

HMF: Hydroxymethylfurfural; n.i., not identified. According to the European Honey Legislation [121] and the Codex Alimentarius Standards [122], honey moisture should not exceed 20%, with the exception of heather (*Calluna* sp.), which should not surpass 23–25%, acidity, should be less than 50 milli-equivalents (MEQ) acid/kg, with glucose and fructose composition not less than 60 g/100 g and HMF not more than 40 mg/kg, except honeys from tropical climates which should not be more than 80 mg/kg. As it can be foreseen, in some monofloral honeys, the acidity surpasses 50 MEQ/kg, possibly indicating the presence of undesirable fermentations, such as: *Arbutus* sp*., Ceratonia* sp. and *Thymus* sp. from Portugal. Based on their botanical origin, nectar honeys have low ash content compared to honeydew honey.

Generally, the moisture content is an indicator of honey’s freshness, being useful to improve its conservation, storage and prevent the growth of moulds (*Penicilium* and *Mucors*). The growth of mould gives an off-taste due to the growth levels of ethanol, butanediol and glycerol, which reduce the products quality. Furthermore, the moisture content is a useful parameter in describing the viscosity and moistness of honey. As previously stated, in Codex Alimentarius (2001) [122] and EU Council directives [121], the maximum moisture content value of *Erica* monofloral honey should not exceed 23% and 20% in other monofloral honeys. The moisture content varies depending on external factors, such as environment and harvesting period, and internal factors such as the maturity reached in the hive [123]. In most blossom honeys, fructose and glucose represent the great majority of honey sugars. According to Codex Alimentarius, the sum of fructose and glucose must be more than 60% for nectar honey and 45% for honeydew honey. The sucrose content should not exceed 5%, with few exceptions such as the Lavender honey. As can be seen in Table 3, the HMF exceeded 40 mg/kg in the monofloral honeys of *Brassica sp.* (Bulgaria), *Calluna sp*. (Turkey), *Ceratonia sp*. and *Echium sp*. (Portugal). Several studies reported slightly higher moisture content in *Calluna sp*. (Turkey), *Erica sp*. (Portugal) and *H. annus* (Romania). Comparatively, the physicochemical characteristics of monofloral honeys originating from Spain were in accordance with the indicated international standards. Bentabol Manzanares et al., (2014) [124] determined the physicochemical characteristics of heather, avocado, oregano, barilla (*Messembryanthemum crystallinum*), malpica (*Carlina xeranthemoides*), poleo (*Bystropogon origanifolius*), relinchón (*Hirstfeldia incana*) and tedera (*Aspalthium bituminosum*) in monofloral honeys. The lowest moisture content (15.1 g/100 g) is noticed in relinchón honey and the highest value (18.4 g/100 g) in barrilla honey, whereas the moisture of heather (17.24 g/100 g) is similar to those reported by other researchers [45,46,104]. Furthermore, the HMF ranged between 5.36–15.00 (mg/kg) in Barilla and Oregano honeys, respectively.

In the study conducted by Bouhlali et al., 2019 [50], the water content of the monofloral honey present in Morroco was found to be from 14.55 to 20.99%, the water activity level varied between 0.49 and 0.58%, whereas the total acidity (directly associated to pH and free acidity) had values between 23.55 and 43.53 meq/kg. Furthermore, the highest fructose–glucose ratio (>1.5) belonged to eucalyptus and reseda honey samples.

Due to handling and processing inconveniences, honey has the tendency to crystallize. To avoid this, thermal processing is applied to avoid crystal formation, as well as microbial contamination [125]. Nonetheless, thermal treatment is followed by undesirable consequences, such as colour changing or browning of honey, but also the formation of HMF, an important quality parameter of honey. It is an indicator of honey freshness and overheating of honey leads to conversion in the cytotoxic and mutagenic compound 5-sulfoxymethyfurfural, which can also be generated under inadequate handling and storage conditions (temperature, pH, moisture) [126]. In fresh honeys there is practically no HMF, or it is in very low content; however, it increases upon storage [127]. Council Directive (2001/110/CE) [121] demands a maximum of 40 mg/kg of HMF in honey. The proposal for a higher maximum value of HMF (80 mg/kg) in some countries is based on the experience that HMF increases in honey stored in warm climate countries.

In a recent study, Park et al., (2020) [119] evaluated the physicochemical properties, mineral content and antibacterial activity of Hovenia monofloral honey. Their results showed that Hovenia monofloral honey was composed of glucose (29.0 ± 0.42%), fructose (35.9 ± 0.78%), moisture (18.9 ± 0.28%), reducing sugar (64.9 ± 0.35%), sucrose (3.9 ± 1.63%), ash (0.1 ± 0.00%) and HMF (0.00 ± 0.00%).

Currently one of most innovative commercial products available on the market is Manuka honey [128], which is a monofloral dark honey. The chemical composition and the variety of beneficial nutritional and health effects of manuka honey are revised by Alvarez–Suarez et al. (2014) [55]. It is derived from the manuka tree *Leptospermum scoparium* (*Myrtaceae* family). The manuka tree grows as a shrub or a small tree in New Zealand and eastern Australia [56]. This type of honey has attracted the attention of several researchers for its biological properties, especially for its antimicrobial and antioxidant activity [55].

The composition of Manuka honey is mainly of carbohydrates, mineral elements, proteins, fatty acids and phenolic and flavonoid compounds. One of the major antibacterial components of Manuka honey is methylglyoxal (MGO) [129]. Sugars are the main components in Manuka honey. The authors identified fructose, glucose, sucrose and maltose in all honey samples. Fructose and glucose are the main monosaccharides. The content of fructose is higher compared to the glucose content. The content of fructose plus glucose of manuka honey (60.7 g/100 g) was similar to that reported by Moniruzzaman et al. (2013a) [130]. The sum of fructose and glucose in Manuka honeys is higher than 60%, which is in accordance with Codex Alimentarius (2001).

Methylglyoxal is also found in *Leptospermum scoparium* (manuka) and *Kunzea ericoides* (kanuka) honeys. Manuka honey contains high concentrations of trimethoxybenzoic acid and methylglyoxal, whreas Kanuka honey contains a high concentration of methoxyphenyllactic acid. The phenolic components increase with maturation in both honey types [90]. According to Beitlich et al. (2014) [131], nonvolatiles profiles of manuka honey show leptosin, acetyl-2-hydroxy-4-(2-methoxyphenyl)-4-oxobutanate, 3-hydroxy-1-(2-methoxyphenyl)-penta-1,4-dione, kojic acid and 5-methyl-3-furancarboxylic acid as predominant. Kanuka honey is characterized by the folowing nonvolatiles compounds: 4-methoxyphenyllactic acid, methyl syringate, p-anisic acid and lumichrome, whereas volatile compounds for manuka honey are 2-Methylbenzofuran, 2′-hydroxyacetophenone and 2′-methoxyacetophenone, whereas kanuka honey was characterized by 2,6,6-trimethyl-2-cyclohexene-1,4-dione, phenethyl alcohol and p-anisaldehyde.

Manuka honey has a relatively low pH (about 3.5–4.5), which contributes to the inhibition of microbial growth [132]. Furthermore, the low levels of wound pH can reduce protease activity and increase fibroblast activity and wound healing [133,134].

According to Kato et al. (2012) [56], a novel glycoside, leptosin is a characteristic compound of manuka honey. Furthermore, as this type of honey has a low (11,59%) moisture content [130], it allows a long-time protection against microbial attacks [131]. Honey from *Koompassia excels* (Tualang) contains more free radical scavenging, antioxidant activity, flavonoids and phenolic acids, and its health benefits are better compared to other local Malaysian honeys, such as Manuka honey [132]. According to Mohamed et al. (2010) [53], the tualang-derived honey has good colour intensity and contains phenolic compounds that possess relatively good antioxidant activity.

## 6. Major Mineral Composition of Some Monofloral Honey

Generally, the mineral content in honey is low ranging between 0.02 and 0.3%. It is influenced by the nectars chemical composition which varies according to soil composition (geographical origin) and floral type (botanical origin). Variations of soil and climatic conditions may also influence the mineral composition, as well as the extraction method and harvesting techniques [135,136,137].

According to the International Honey Commision (2009) [137], requirements in individual countries include parameters such as diastase activity (general, no less than eight on the Schade scale), electrical conductivity (e.g., in honeydew honey, no less than 0.8 mS/cm), HMF (general, no more than 40 mg/kg), free acidity (in general, no more than 50 milli-equivalents acid/1000 g), proline (no less than 25 mg/100 g) and pH, insoluble impurities (no more than 0.1 g/100 g).

The most significant minerals in honeys are Potassium (K), Magnesium (Mg), Calcium (Ca) and Sodium (Na). Less abundant elements are Iron (Fe), Copper (Cu), Manganese (Mn) and Chlorine (Cl), followed by trace elements of Borron (B), Phosphorous (Ph), Sulfur (S), Silicon (Si) and Nickel (Ni), among others [138]. Due to its fast secretion by nectar sources, K is the main element, reaching almost 80% of the total content [137]. Multiple studies have been conducted to classify monofloral honeys by their mineral composition [139,140,141,142,143].

Honey has also been regarded as a potential environment pollution indicator, as a result of a bio-accumulative process in the borders of urban and industrial areas, as well as in extra-urban crossroads, where traces of some mineral compounds and/or heavy metals were found [144].

As can be seen in Table 4, the monofloral honeys from Spain are abundant in K, Ca and Mg, ranging between 2.70–530.0, 23.00–387.0 and 41.0–331.0 mg kg^−1^ respectively. High discrepancy was noticed in the case of Na content, ranging between 9.18 and 1321.40 mg kg^−1^. The honeys from Portugal exhibited high potassium content, accounting for 76% with an average content of 1150.1 mg g^−1^. Except for the honeys from Spain, which exhibited high amounts of Na, research from other countries revealed K to be the most abundant mineral. Furthermore, from the monofloral samples of Morocco, K was the predominant mineral, followed by Na, Ca and Mg, which were found in the highest amount in carob honey [50], whereas the mineral contents of Hovenia monofloral honey included Ca (20.1 ± 1.06 mg L^−1^), K (407.5 ± 3.11 mg L^−1^), P (20.6 ± 1.77 mg L^−1^), Mg (10.7 ± 1.1 mg L^−1^), Na (1.8 ± 0.28 mg L^−1^), and Zn (Zinc) (13.9 ± 15.1 mg L^−1^).

In their extensive review, Solayman et al., 2015 [153] reported detailed information regarding the physicochemical properties of honey, particularly the mineral and heavy metal contents, as well as their medicinal properties.

## 7. Antioxidant Activity of Monofloral Honeys

Phenolic compounds are mostly divided into two main groups: flavonoids and phenolic acids. Honey contains a significant number of polyphenolic compounds, widely used as plant origin and honey quality indicators. The total phenolic content (TPC) and total flavonoid content (TFC) are well-known criteria for the initial assessment of the bioavailability of honey and other bee products.

Worldwide, multiple studies assessed the antioxidant activity of monofloral honeys. This research is often accompanied by the measure of some spectrophotometric parameters such as TPC and TFC, as well as the electrical conductivity and/or colour. In fewer cases, these studies are accompanied by additional characteristics, such as: melissopalynological analysis, chromatographic phenolic profile or mineral composition of major and trace elements. In the last decade, multiple studies have evaluated the antimicrobial, antioxidants and radical scavenging properties of monofloral honeys from different geographical origin, such as: Burkina Fasan, Hungary, Italy, Poland, Portugal, Turkey and Malaysia. Table 5 summarises a selection of monofloral honeys’ antioxidant activity.

As can be seen, the Portuguese honeys’ TPC (mg GAE/100 g) varies from 31.85 in Lavender to 117.65 in Strawberry tree and Heather honeys. The flavonoid content (mg of QE/100 g) ranges between 1.93 in Sunflower to 21.16 in Heather honey. In the study performed by Rosa and his collaborators (2011) [195], it was found that Strawberry tree honey had the highest TPC, as well as higher activity in the DPPH and FRAP tests, compared to Citrus, Heather and Eucalyptus honeys. Furthermore, from the total phenols content, homogentisic acid (i.e., the chemical marker for strawberry tree honey) was more than 60% and showed significant antioxidant and antiradical activities, as well as a protective effect against thermal cholesterol degradation. Wang et al. (2019) [196] determined the TPC and TFC from *Prunella vulgaris* (PVH) monofloral honey, a known traditional Chinese medicine, which displayed a TPC of 145.7 mg chlorogenic acid equivalent (CAE)/kg and a TFC of 10.0 mg QE/kg. The TFC of PVH was higher compared to other monofloral honeys with potential medicinal value, such as Acacia, Buckwheat and Manuka, but lower compared to Heather, Sunflower, Canola, Pine, and Thyme. Additionally, the TFC was relatively high compared to *Rhododendron* and Chestnut from Turkey and coffee honey from Brazil. Furthermore, low TPC in the *Rhododendron* honey was noticed in the samples from Ordu and Artvin, whereas the samples from Zonguldak and Kastamonu exhibited the highest TPC compared to the other regions. Therefore, the TPC is strongly correlated to the antioxidant activity, as demonstrated in multiple studies. In the study conducted by Socha et al., 2011 [197], the TPC in Polish honeys was much lower (4.46–15.04 mg GAE/100 g), especially in Buckwheat honey (15.04 mg GAE/100 g).

Kus et al., 2014b [87] evaluated the TPC and antioxidant activities of several polish honeys. Their results showed that the TPC ranged between 121.6–1173.8 mg GAE/kg. Similar phenolic contents were reported for Mexican honeys (283.9–1142.9 mg GAE/kg), from which the highest TP content was found in multifloral, orange blossom and bell flower samples [198]. Furthermore, TPC was found to be similar in Burkina Fasan honeys (325.9–1147.5 mg GAE/kg), with the richest content in honeydew and *Vitellaria* honeys [154]. In the study conducted by Anand et al., 2018 [159], the lowest TPC was recorded for *Agastache* honey (853.6 ± 5 µg GAE/g) and the highest for Jelly bush honey (1415.6 ± 126 µg GAE/g). Jelly bush honey contained the highest TFC (53.9 ± 10.9 µg CE/g), whereas Tea-tree honey had the lowest TFC (20 ± 4.3 µg CE/g). The scavenging activity of free radicals determined by DPPH assay indicated that the highest antioxidant activity was present in Manuka honey (18.69 ± 0.9 µmol TE/g) followed by Jelly bush and Tea-tree. Similarly, the scavenging activity of free radicals determined by TEAC assay indicated that the highest activity was shown in Manuka honey (30.72 ± 0.27 µmol TE/g), followed by *Agastache* and Jelly bush.

Multiple studies have demonstrated the potential therapeutic properties of monofloral honeys due to their bioactive compounds, as well as based on their geographical origin. Further details can be seen in Table 5.

In the last decade, monofloral honeys have attracted great interest from researchers. For the first time, the phytochemical composition of several significant monofloral Cuban honeys and their relationships with the biological activities were studied by Alvarez–Suarez et al. Their antioxidant [199] and antimicrobial [200] properties were evaluated and discussed also in terms of correlation with amino acids, proteins, carotenoids, colour, TPC and TFC. The analysed samples possessed important antioxidant and antibacterial properties, as well as high concentrations of phenolic acids, flavonoids and carotenoids. Later, Alvarez–Suarez et al., 2012 [66] showed that Cuban honeys ether-soluble phenolic fraction exhibits significant radical scavenging activity and protection of RBCs against hemolysis and lipid peroxidation induced by free radicals, as well as protection against depletion of SOD and GSH enzymes.

In the study conducted by Park et al. 2020 [119], the antioxidant activity of Hovenia honey was evaluated. Their study showed that the Hovenia honeys had a significantly (*p* < 0.05) higher DPPH radical scavenger activity (36.3–38.7 μmol TE/100 g honey) and a similar ABTS radical scavenger activity (129.5–141.9 μmol TE/100 g honey), compared to Acacia honey (ABTS: 13.9–22.4 μmol TE/100 g honey and DPPH: 130.6 ± 6.74 μmol TE/100 g honey). Interestingly, the amounts of TPC and TFC were not significantly different between these honey types.

These findings suggest that beside floral origin, the provenance is also significant for the phenols content of honeys from the same species.

## 8. Health Effects of Monofloral Honeys

Honey has been widely used as a folk medicine, being acknowledged centuries ago [201,202]. Hippocrates emphasized the similarity of honey to air and water, that honey is a very valuable antidote and that it can be used alone or mixed with herbs in the form of sherbet, ointment, and drug in the treatment of many physical and mental illnesses [203]. With the widespread use of apitherapy in recent years, the use of bee products and honey has increased [204]. In Table 6, several in vivo studies on animal models and human subjects are summarized based on honey types, antioxidant, anti-diabetic, anticancer and wound healing properties.

### 8.1. Wound Healing

Since ancient times, honey has been used for the treatment of wounds. The discovery of antibiotics has led to a decrease in honey use, but the development of antibiotic resistance increased the interest in honey again due to its antibacterial properties. However, attention should be paid to the quality of honey used in medical practices. Plants in honey harvest areas are exposed to herbicides and pesticides, industrial heavy metals and environmental pollution contaminated with antibiotics, and, as a result, toxic compounds can be found in honey. In addition, the harmful microbial load of honey is ignored. The content and standards of honey used especially for health purposes should be determined [227]. There is evidence that honey can heal partial thickness burns and postoperative infected wounds more quickly and effectively than conventional treatments [228]. Recently, honey has been extensively applied in wound treatment as an alternative to more expensive and advanced wound products. It reduces redness, swelling and pain and, when applied, there is usually no discomfort other than a slight stinging sensation [229].

### 8.2. Antioxidant Activity

As exemplified in the previous sections, monofloral honey is rich in both enzymatic and non-enzymatic antioxidants, polyphenols and minerals. In the study conducted by Omotayo et al. (2010) [205], it was investigated whether tualang honey could reduce hyperglycemia and ameliorate oxidative stress in streptozotocin-induced diabetic rats. The honey-treated groups showed an increase activity of catalase, glutathione peroxidase, glutathione reductase and glutathione-S-transferase compared to control. The same was noticed in the study of Erejuwa et al. [206], which showed that the combination of glibenclamide, metformin and taulang honey significantly up-regulated CAT activity, reduced TBARS levels and down-regulated GPx activity.

Sibel et al. [208] showed that Rhododendron honey treatments showed an antioxidant effect on blood plasma and organ tissues Thus, a significant increase in GTX was shown at a high dose of mad honey and decreased levels of MDA levels in the group administered a low dose of honey (0.1 g/kg/bw). Furthermore, the honey-administered group, at low doses of honey, was closely related to the control group in lung, heart, spleen, testicle and epididymis tissues and medium doses of honey in the testicle and epididymis.

Eraslan et al. [209] demonstrated that the administration of pine honey alleviated the tissue MDA levels and CAT, SOD and GSH-Px activities in the trichlorfon-administered group. Thus, they demonstrated that, in order to minimize the risk of foodborne trichlorfon intoxication, as well as its adverse effects, pine honey may be safely included in the daily human diet. In a different study, Bezerra et al. [210] showed the positive effect of malicia honey on the lipid metabolism, antioxidant status and intestinal health of rats with diet-induced dyslipidaemia. The dyslipidaemia group that received honey showed lower values of GPx and higher MDA levels.

### 8.3. Anti-Obesity

Honey has the potential to control obesity by reducing excess weight gain and other obesity parameters such as triglyceride levels. However, its effects on cells that store lipids (adipocytes) are still unclear. In the study conducted in order to observe the effects of pineapple honey on adiposity growth and lipid accumulation in vitro, it was determined that pineapple honey had a total phenolic content of 0.0379 ± 0.001 mg/100 mL GAE and a total flavonoid content of 0.098 ± 0.001 mg catechin/kg. It has been found to significantly inhibit the proliferation of adipocytes starting at 6.25% of the pineapple honey concentration. In addition, honey was found to significantly reduce lipid droplet size between 33.78% and 70.36%, compared with the control [184].

The influence of Clover honey on weight gain, adiposity and blood lipid profile on Sprague–Dawley rats has been evaluated [213]. They were equally divided into two groups and provided either clover honey or sucrose. After 33 days, for the honey-fed group, the bw was 14.7% lower, alongside a 13.3% lower consumption of food. Compared to control, the epididymal fat weight was 20.1% lower, as well as the serum concentrations of triglycerides (29.6%) and leptin (21.6%). Their results clearly demonstrated that, in comparison with sucrose, honey reduced weight gain and adiposity, due to lower food intake, as well as promoted lower serum triglycerides.

In a different aspect, the high fat diet rats fed with Gelam and Acacia honeys had lower consumption of food, as well as a lower adiposity index compared to the high fat diet group. Furthermore, rats fed with Acacia honey showed a significant increase in the relative organ weight compared to the control group, particularly the liver, heart and lung [215]. Even though, in the experimental diabetes mellitus, the supplementation with tualang honey resulted in weight gain of diabetic rats, its supplementation in human (especially obese) diabetic patients may necessitate a dose adjustment and reduced calorie intake [205].

### 8.4. Gastrointestinal Protective Effects

Honey produced from medicinal plants is generally a great hope for human health. The chemical composition and gastrointestinal protective effects of a new monofloral honey from *Prunella vulgaris* (PVH) have been identified, and it has been reported to provide basic data on PVH for future applications supporting the prevention of colitis [148]. Furthermore, Rhododendron honey is used as an alternative treatment for gastritis, stomach ulcer, constipation, hypertension, coronary heart disease and impotence [229].

Kocyigit et al. [183] showed that, due to its high phenolic and antioxidant contents, *Quercus pyrenaica* honeydew honey were tested against gastric cancer cells.

Honeydew honey had proliferative effects due to its antioxidant activity, and high concentrations showed cytotoxic, genotoxic, and apoptotic effects in the tested cancer cells. All these effects were higher with *Quercus* application compared with that of polyfloral honey, which possessed lower phenolic content. Their results demonstrated that honeydew honey may contribute to the future development of therapeutics in patient’s suffering from several cancers.

Oral pretreatment with Chestnut honey (2 g/kg), once daily for seven consecutive days, prevented indomethacin-induced gastric lesions in rats, reduced the ulcer index, microvascular permeability and stomach’s myeloperoxidase activity [212].

### 8.5. Anti-Fatigue and Antidepressant Effects

It has been stated that honey has a fatigue-relieving and soothing quality due to its alkaline balance. Honey, which also affects the nervous system, is good for headaches, insomnia and depression with its antidepressant and sedative properties [230].

### 8.6. Antibacterial and Antimicrobial Activity

Honey provides benefits against *Streptococcus mutans* infections, dental plaque and caries and gingivitis and bad breath, and also contributes to the prevention of side effects associated with the treatment of head and neck cancers, namely radiation-induced mucositis, xerostomia and poor wound healing [231,232]. Methylglyoxal (MGO) is the main antimicrobial marker associated with the use of Mānuka honey as a topical dressing. It is predicted that MGO derived from Mānuka honey may play a role in the enhancement of microbial detection by MAIT cells, which may help MAIT cells (Mucosal-associated invariant T cells) to control microbial infection and systemic immune homeostasis [233]. A recent study showed the differences between the antibacterial effects of Hovenia and Acacia honeys [153]. The minimum inhibitory activity of Hovenia and Acacia honeys was evaluated against gram positive (*S. aureus* and *L. monocytogenes*) and gram negative (*E. coli* O157:H7 and *S. typhimurium*) bacteria. Their results showed similar MIC for both honeys, as follows: 25–50% (*w*/*v*) against *E. coli* O157:H7 and *S. typhimurium* and 25% (*w*/*v*) against *S. aureus* and *L. monocytogenes*. Furthermore, the MIC values of artificial honey (sugar constituted) against foodborne bacteria exceeded 50% (*w*/*v*), indicating that a part of antibacterial activity of Hovenia and Acacia monofloral honeys was derived from the TPC and TFC.

Moghadam and Khaledi (2021) [234] studied the minimum inhibitory concentration and the minimum bactericidal concentration of six Iranian honey samples in comparison with Manuka honey against reference strains of *Staphylococcus aureus* (ATCC 29737), *Pseudomonas aeruginosa* (ATCC 27853), *Klebsiella pneumonia* (ATCC 10031) and *Escherichia coli* (ATCC 10536). Their results showed high antimicrobial and anti-biofilm activity in Iranian honeys compared to Manuka honey.

Majtan and Majtan (2010) [235] studied the antibacterial properties of Slovak honey types such as Acacia, Canola, Meadow and Forest, compared to Manuka honey. The results showed that forest honey had an inhibitory activity similar to that of manuka honey for some bacteria. Forest honey is more effective against *Proteus* sp. and *Pseudomonas aeruginosa* than manuka honey. In recent years, increased bacterial resistance to a lot of antibiotics and the various complications with chronic wounds are presented. Boateng and Diunase (2015) [128] compared Cameroonian honeys in the respect of physicochemical, antioxidant and antibacterial activity (that may contribute to the functional wound healing) to those of Manuka honey.

### 8.7. Antidiabetic Effects

Multiple reports have demonstrated the positive role of honey in regulating the blood glucose level. However, due to its high sugar content, honey is considered detrimental to diabetics. As stated in Section 7, fructose content in monofloral honey varies from 23% in Eucalyptus to 45% in Heather, followed by Fennel with 44.9%. Therefore, its high fructose content along with minerals, phenolic acids and flavonoids has a role in regulating blood glucose levels. Furthermore, Omotayo et al., 2010 [205] showed that consumption of tualang honey significantly decreased the elevated levels of TBARS in streptozotocin-induced diabetic rats, showing that the honey-treated diabetic rats had reduced lipid oxidative damage.

In a recent study by Whitfield et al. [220], the mixture of Kanuka honey with chromium, cinnamon and magnesium was studied for its effect on lipid profile, glycaemic control and weight in 12 patients with type 2 diabetes. Based on their results, consumption of the 53.5 g honey mixture for 40 days significantly increased the bw and improved lipid parameters in the subjects. Additionally, susceptibility in the increase of HDL and reduction of systolic blood pressure was also noticed. Finally, the honey mixture did not affect glycaemic control and the metabolism of glucose.

### 8.8. In Vitro Studies Correlated to Polyphenols

Many of the polyphenols found in honey, such as caffeic acid, caffeic acid phenyl esters, chrysin, galangin, quercetin, kaempferol, acacetin, pinocembrin, pinobanksin and apigenin have potential in the treatment of cancer. Jaganathan and Mandal (2009) [236] reviewed the antiproliferative effect of polyphenols from honey in various cancer cell lines. Manuka honey has an antiproliferative activity on three different cancer cell lines: murine melanoma (B16.F1), colorectal carcinoma (CT26) and human breast cancer (MCF-7) cells in vitro. It is effective at concentrations as low as 0.6% (*w*/*v*) [182]. Alvarez Suarez et al. 2012 [66] studied the protective effect of such unifloral honeys against lipid peroxidation in an in vitro model of rat liver homogenates and the ability of the phenolic extracts of *Turbina corymbosa* L. (i.e., Christmas vine) and *Gouania polygama* (Linen vine) honeys to inhibit the oxidative damage induced by the 2,2′-azobis(2-methylpropionamidine) dihydrochloride in erythrocytes.

Tsiapara et al. (2009) [114] presented antiprolifertive effects from Greek honey extracts from thyme, pine and fir honey on breast cancer (MCF-7), prostate cancer (PC-3) and endometrial cancer (Ishikawa) cells. Thyme honey reduced the viability of Ishikawa and PC-3 cells. They concluded that Greek honeys are rich in phenolic compounds and may prevent cancer-related processes in breast, prostate and endometrial cancer cells.

In another study, Jaganathan (2012) [237] examined the apoptotic effect of caffeic acid, one of the phenolic constituents of honey, in HCT 15 colon cancer cells. The author promoted the caffeic acid as a candidate in the chemoprevention of colon cancer. The caffeic acid inhibited the colon cancer cell proliferation in a dose-dependent manner. The antiproliferative effect of caffeic acid was estimated using 3-(4,5-dimethylthiazol-2-yl)-2,5-diphenyl tetrazolium bromide (MTT) assay. Swellam et al. (2003) [238] examined the effect of pure unfractionated honey in three human bladder cancer cell lines (T24, 253J and RT4) and in one murine bladder cell line (MBT-2) using MTT assay. The authors received significant inhibition of the proliferation of T24 and MBT-2 cell lines by 1–25% honey and of RT4 and 253J cell lines by 6–25% honey.

Pichichero et al. (2010) [161] investigated the antiproliferative effect of honey or chrysin (5,7-dihydroxyflavone) on human (A375) and murine (B16-F1) melanoma cell lines with MTT assay and Trypan Blue Exclusion Test. Chrysin is a natural flavone found in Acacia honey. Both tests showed that these compounds can induce an antiproliferative effect on melanoma cells. The same authors concluded that the antiproliferative effects of honey are due to the presence of chrysin. In this respect, chrysin is a potential candidate in the prevention and treatment of cancer. Aliyu et al. (2012) [155] provided evidence on the apoptotic role of Acacia honey from Pakistan on the PC-3 Human prostate cancer cell line. The possible mechanism of this process is the modulation of pro-inflammatory cytokines and regulation of prostate specific antigen in vitro.

The antitumor effects of honey on liver cancer cells have been reported by Aziz Baiomy et al. (2009) [239]. The authors reported that honey extracts exerted cytotoxic, antimetastatic and anti-angiogenic effects in HepG2 cells.

Anticancer activity of honey has been proved against various cancer cell lines and tissues in animals and humans [240], and multiple reviews summarise the anticancerogenic properties of different honey types and components of honey [16,241,242,243]. Tualang honey is a Malaysian multifloral jungle honey with many potential health benefits such as antimicrobial, anti-inflammatory, antioxidant, antimutagenic, antitumor, antidiabetic and wound-healing properties. Some of its properties are similar to Manuka honey. The differences include higher phenolics and flavonoids in Tualang honey. Tualang honey is also more effective against some gram-negative bacterial strains in burn wounds compared to the Manuka honey. The honey is produced by *Apis dorsata*, which builds hives on branches of tall Tualang trees located mainly in Peninsular Malaysia [16].

Manuka honey, even at lower concentrations of 0.6% (*w/v*), inhibited the cell proliferation in multiple cell lines (human breast cancer MCF-7, murine melanoma B16.F1 and mouse colon carcinoma CT26) in a dose and time dependent manner [182].

In a recent study, Abd Kadir et al., (2013) [207] studdied the inhibitory effect of Malaysian tualang honey on the development of 7,12-dimethylbenz(α)anthracene (DMBA)-induced breast cancer in rats. Their results showed that the untreated DMBA-induced breast cancer rats (control rats) developed tumors earlier compared to the honey-treated DMBA-induced breast cancer rats. The control rats also showed a marked increase in tumor size over a shorter period. On the contrary, a reduction in the growth and size were noticeably reduced in the honey-treated DMBA-induced breast cancer rats compared to the untreated cancer rats. Furthermore, a lower number of tumors were noticed in the honey-treated rats compared to the controls. Even though not statistically significant, an increase in apoptotic index was noticed by higher honey doses. Higher grade tumours were observed in the untreated rats, compared to lower or medium grade ones reported in the honey-treated group. The histological analysis also revealed that the cancer cells from the honey-treated rats were more identical, with denser nuclei, while those of the control rats had more pleomorphic cells with more prominent nuclei. Finally, lower prominent vasculature surrounding the tumor nodules and significantly reduced weight and volume in the tumor mass were noticed (paler, smaller and softer with necrosis spots) in the honey-treated group.

## 9. Conclusions

Numerous honey compounds act as natural antioxidants. From ancient times to nowadays, honey has had a potential role in contributing to human health. Bee honey is chemically very complex, and the chemical composition strongly depends on its botanical origin. Monofloral honeys are also an economic income source for many countries.

The locally available honey types in different countries are rich in bioactive components displaying excellent applications for human health. There are approximately 200 compounds in honey. The variety of these compounds results in different color, taste, honey type and therapeutic activities. Honey might be utilized as a potential source of natural antioxidants. Furthermore, the consumption of honey has high nutritional and therapeutic values. The concentration of different compounds in honey depends mainly on various factors, such as floral source and honey type, as well as environmental and processing factors. These factors affect the biological activities of each type of honey in the world.

In brief, monofloral honeys present a variety of dietary phytochemical compounds with functional properties such as phenolic acids, minerals, phenolics and flavonoids. Therefore, considering the food industry and the positive effects on human health, monofloral honeys have tremendous potential for the production and use as natural and functional ingredients with special attention to their use in the medical field. To our knowledge, this is the first study of the antioxidant properties, mineral composition and health properties of monofloral honey types.

## Figures and Tables

**Table 1:** Monofloral honeys taxa based on provenance.

Geographical Origin/Provenance	Monofloral Honey Taxa			Reference
	Family	Specie	Common Name	
**Turkey**	Apiaceae	*Pimpinella* sp.	Anise	[22,23,24,25,26,27,28,29,30,31]
	Asteraceae	*Centaurea solstitialis* L.	Yellow star-thistle	
		*Centaurea cyanus* L.	Cornflower or bachelor’s button	
		*Helianthus annuus* L	Common sunflower	
		*Taraxacum farinosum* Hausskn. & Bornm. Ex Hand.-Mazz.	Turkish cırtlık	
	Brassicaceae	*Brasssica* sp.	Canola	
	Ericaceae	*Calluna vulgaris* (L.) Hull	Common heather	
		*Rhododendron sp.*	Rhododendron	
	Fabaceae	*Astragalus microcephalus* Willd	Milkvetch	
		*Robinia pseudoacacia* L.	Black locust	
		*Trifolium sp.*	Clover	
		*Vicia cracca* L.	Tufted vetch or blue vetch	
	Fagaceae	*Castanea sativa Mill.*	Chestnut	
		*Quercus cerris L.*	Turkey oak or Austrian oak	
	Lamiaceae	*Thymus* sp.	Thyme	
		*Vitex agnus-castus* L.	Vitex or chaste tree	
		*Lavandula stoechas* L.	Spanish lavender or topped lavender	
		*Lavandula pedunculata* Mill.	French lavender	
	Malvaceae	*Gossypium barbadense* L.	Sea island cotton	
		*Gossypium hirsutum* L.,	Upland cotton or Mexican cotton	
		*Tilia tomentosa* Moensch	Silver linden	
	Pinaceae	*Cedrus* sp.	Cedar	
		*Pinus brutia* Ten.	Turkish pine	
	Rhamnaceae	*Paliurus spina-christi* Mill.	Jerusalem thorn	
	Rosaceae	*Prunuscerasus* L.	Sour cherry	
	Rutaceae	*Citrus* sp.	Citrus	
**Bulgaria**	Apiaceae	*Coriandrum sativum* L., *Daucus* sp.		[32,33,34,35]
	Asteraceae	*Carduus nutans* L.	Musk thistle or nodding thistle	
		*Centaurea cyanus* L.		
		*Helianthus annuus* L,		
		*Onopordum acanthium* L.	Cotton thistle	
	Brassicaceae	*B. napus*		
	Fabaceae	*R. pseudoacacia*, *Styphnolobium japonicum* (L.) Schott	Japanese pagoda tree	
		*Vicia sp.*		
	Honeydew honey	Forest honey		
	Polygonaceae	*Fagopyrum esculentum* Moench	Common buckwheat	
	Rosaceae	*Prunus sp.*		
	Salicaceae	*Salix* sp.		
	Saxifragaceae	*Saxifraga adscendens* L.	Wedgeleaf saxifrage	
	Tiliaceae	*T. cordata*		
**Romania**	Asteraceae	*H. annuus*, *Taraxacum officinale* (L.) Weber ex F.H.Wigg.	Common dandelion	[36]
	Betulaceae	*Corylus avellana* L.	Common hazel	
	Brassicaceae	*Brassica napus* L.	Canola	
	Cornaceae	*Cornus mas* L.	European cornel or Cornelian cherry	
	Fabaceae	*R. pseudoacacia*		
	Fagaceae	*C. sativa*		
	Lamiaceae	*Thymus serpyllum* L.	Breckland thyme or wild thyme	
	Rosaceae	*Rubus idaeus* L.	European red raspberry	
		*Malus domestica* Borkh.		
		*Malus floribunda* Siebold ex Van Houtte	Japanese crabapple or purple chokeberry	
		*Prunus padus* L.	Bird cherry	
	Salicaceae	*Salix caprea* L.	Goat willow	
	Tiliaceae	*Tilia platyphyllos* Scop.	Large-leaved linden	
		*Tilia cordata* Mill.	Small-leaved lime or little-leaf linden	
**Ukraine**	Asteraceae	*H. annuus*		[37,38]
	Brassicaceae	*B.napus*		
	Fabaceae	*R. pseudoacacia*		
	Polygonaceae	*Fagopyrum esculentum* Moench.		
	Tiliaceae	*Tilia platyphyllos* Scop.	Large-leaved lime or large-leaved linden	
**Italy**	Apiaceae	*Coriandrum sativum* L.	Chinese parsley or cilantro	[39,40,41,42,43]
	Asteraceae	*Dittrichia viscosa* (L.) Greuter	False yellowhead	
		*H.annuus*		
	Ericaceae	*Erica arborea* L.	Tree heather	
		*Erica scoparia* L.		
		*Arbutus unedo* L.	Arbutus or strawberry tree	
		*Rhododendron ferrugineum* L.	Snow-rose or rusty-leaved alpenrose	
	Fabaceae	*Trifolium pratense* L.	Red clover	
		*Hedysarum coronarium* L.	French honeysuckle or sulla	
		*Medicago sativa* L.	Alfalfa	
	Fagaceae	*C. sativa*		
	Honeydew honey	*Abies alba* Mill. and/or *Picea abies* L.	Fir honeydew	
		Forest Honeydew, Insect: *Metcalfa pruinosa* Say	Forest honeydew	
	Myrtaceae	*Eucalyptus camaldulensis* Dehnh.	River red gum	
	Rhamnaceae	*Paliurus spina-christi* Mill.	Marruca	
	Rutaceae	*Citrus sp.*		
	Tiliaceae	*R. pseudoacacia* L.		
		*T. cordata*		
	Xanthorrhoeaceae	*Asphodelus microcarpus* Salzm. et Viv.	Asphodel	
**Portugal**	Aamaryllidaceae	*Narcissus triandrus* L. (Angel’s tears)		[44,45,46]
	Ericaceae	*Arbutus unedo* L.,		
		*Erica sp.*	Heather	
		*Erica umbellata* L.	Dwarf Spanish heather	
		*Rhododendron ponticum* L.	Common rhododendron	
	Fabaceae	*Cytisus scoparius* L. (Link)	Common broom or Scotch broom	
		*Ceratonia siliqua* L	Carob	
	Fagaceae	*Fagus sylvatica* L.	European beech	
	Lamiaceae	*Thymus sp.* (thyme),		
		*Lavandula stoechas* L.	Spanish lavender or topped lavender	
		*Lavandula latifolia* Medik.	Portuguese lavender or broadleaved lavender	
	Myrtaceae	*Eucalyptus globulus* Labill.	Southern blue gum	
	Rhamnaceae	*Frangula azorica* Grubov	Azorean buckthorn	
	Rosaceae	*Prunus lusitanica* L.	Portugal laurel	
	Rutaceae	*Citrus sinensis* (L.) Osbeck	Common sweet orange	
	Santalaceae	*Viscum cruciatum* Sieber ex Boiss.	Red-berry mistletoe	
**Hungary**	Amaryllidaceae	*Allium ursinum* L.	Wild garlic	[47,48,49]
	Apocynaceae	*Asclepias* sp.	Milkweeds	
	Apiaceae	*Coriandrum sativum* L.,		
		*Daucus spp.*		
	Asteraceae	*H. annuus*		
		*Solidago* sp.		
	Boraginaceae	*Phacelia tanacetifolia* Benth	Lacy phacelia, blue tansy or purple tansy	
	Brassicaceae	*B. napus*		
	Honeydew honey	Forest Honeydew		
	Fabaceae	*Vicia sp.*		
	Fagaceae	*C. sativa*		
	Rosaceae	*Crataegus sp.*	Hawthorn	
	Tiliaceae	*R. pseudoacacia*		
		*T. cordata*		
**Morocco**	Euphorbiaceae	*Euphorbia resinifera* O. Berg.	Resin spurge	[50,51,52]
	Fabaceae	*Acacia raddiana* Savi	Twisted acacia	
	Lamiaceae	*Lavandula sp.*; *Thymus sp.*		
	Lamiaceae	*Rosmarinus officinalis* L.	Rosemary	
	Leguminosae	*Ceratonia siliqua* L.	Carob	
	Myrtaceae	*Eucalyptus sp.*		
	Nitrariaceae	*Peganum harmala* L.	Harmal or wild rue	
	Resedaceae	*Reseda villosa* Coss.	Mignonette	
	Rhamnaceae	*Zizipus jujuba* Mill	Jujube or Chinese date	
	Rutaceae	*Citrus reticulate* Blanco	Mandarin orange	
**Malaysia**	Fabaceae	*Koompassia excelsa* (Becc.) Taub	Tualang	[53,54]
		*Acacia* sp.		
	Myrtacaeae	*Melaleuca cajuputi* Powell	Gelam	
**New Zealand**	Asteraceae	*Carduus nutans* L.	Musk thistle	[55,56,57,58]
	Boraginaceae	*Echium vulgare* L.	Viper’s bugloss or blueweed	
	Cunoniaceae	*Weinmannia racemosa* L.f.	Kāmahi	
	Fabaceae	*Trifolium* sp.		
	Lamiaceae	*Thymus vulgaris* L.	German thyme	
	Myrtaceae	*Leptospermum scoparium* J.R.Forst. & G.Forst.	Manuka or New Zealand teatree	
		*Metrosideros umbellate* Cav.	Southern rata	
	Proteaceae	*Knightia excelsa* R. Br.	Rewarewa	
	Strasburgeriaceae	*Ixerba brexioides* A.Cunn.	Tawari and Whakou when in flower	

**Table 2:** Phenolic compounds present in monofloral honeys.

Phenolic Compounds	Honey Type	Extraction Technique/Extraction Solvent	Spectral Analysis	Referencces
Flavonols				
Quercetin	Heather, Lavender; Jelly bush (*Leptospermum polygalifolium*), manuka; Clover, Thyme; Liven vine; Christmas vine; Chestnut, Eucalyptus, Citrus, Sulla; Sesame, Coriander, sunflower, Savory, Sage	MeOH; MeCN	RP-HPLC; HPLC-PDA; HPLC-UV; UHPLC-DAD MS/MS	[62,63,64,65]
Quercetin-*O*-rhamnoside	Liven vine	MeOH	HPLC-DAD-ESI-MS/MS	[66]
Quercetin 3-orutinoside (rutin)	Kelulut, Tualang; Chestnut, Eucalyptus, Citrus, Sulla, Sesame	MeOH/water 1:1; MeCN	LC-ESI-MS/MS; HPLC-UV	[64,67,68]
Quercetin 3′,3′-dimethyl eter	Rosemary	MeOH:water (1:1)	CE-ESI-MS	[69]
Quercetin 7,3′-dimethyl eter	Rosemary	MeOH:water (1:1)	CE-ESI-MS	[69]
Quercetin rhamnosyl-hexosyl-rhamnoside	Sulla, Dill, Lemon, Orange	MeOH	HPLC-DAD-MS	[70]
Apigenin	Buckwheat, manuka, tualang, chaste, strawberry tree*; Sesamum indicum*, jujube, longan, Black locust, sunflower, linden, basil, goldenrod, sulla, Citrus, *Ziziphus Spina-Christi,* Kelulut, Tualang; *Sesamum indicum;* Black locust, sunflower, linden, sulla, thistle, basil, buckwheat, rapeseed and goldenrod; *Ziziphus Spina-Christi*; ailanthus, savory; Jujube, longan and chaste	MeOH; MeOH/water 1:1	HPLV-UV; LC-ESI-MS/MS	[64,67,71,72,73,74]
Kaempferol	Buckwheat, manuka, Liven vine; Christmas vine; Rosemary; Tualang, manuka, Black locust, chestnut, savory, sulla, ailanthus, thymus and orange	MeOH; MeOH:water (1:1)	HPLC-DAD-ESI-MS/MS; CE–ESI-MS; LC-ESI-MS/MS	[66,67]
Kaempferid	Rosemary	MeOH:water (1:1)	CE–ESI-MS	[69]
Isorhamnetin	Liven vine; Christmas vine, manuka;	MeOH	HPLC-DAD-ESI-MS/MS	[66,75]
8-methoxykaempferol	Liven vine; Christmas vine	MeOH	HPLC-DAD-ESI-MS/MS	[66]
Galangin	Buckwheat, manuka, black locust, chestnut, savory, sulla, ailanthus, thymus; sage	MeOH; 40/50/10 (*v*/*v*/*v*) water/TFA/MeCN	HPLV-UV; UHPLC-DAD MS/MS	[65,71,76]
Genistein	Acacia, Thymus, Black locust, chestnut, savory, sulla, ailanthus, thymus and orange	MeOH; 40/50/10 (*v*/*v*/*v*) water/TFA/MeCN	HPLC-PDA; HPLC-UV	[75,76,77]
Myricetin	Rosemary; Chestnut, eucalyptus, citrus and sulla; *Sesamum indicum*, black locust, thistle, Lavender, orange blossom, heather; ailanthus, Buckwheat	MeOH:water (1:1); MeOH; MeCN	CE–ESI-MS; HPLC-UV	[64,68,69,73,78]
Methyl anthranilate	Citrus	MeOH	GC–MS	[43]
Flavanols				
Catechin	Kelulut, Tualang; Chestnut, eucalyptus, citrus and sulla; sage; Jujube, longan and chaste; Tualang, pine	MeOH/water 1:1	LC-ESI-MS/MS; HPLC-UV	[67,68,74,75]
Epicatechin	Acacia, Chestnut, eucalyptus, citrus and sulla, manuka, *Ziziphus Spina-Christi*	MeOH	HPLC-UV	[68,79]
Flavanonols				
Pinobanksin	Black locust, Rosemary, manuka, Sulla, Thistle, Citrus, Eucalyptus, Sage; Dill; Jujube, Longan and Chaste	MeOH:water (1:1); MeOH; MeCN;	CE–ESI-MS; HPLC-PDA; UHPLC–UV; UHPLC-DAD MS/MS	[65,69,70,77,80]
Pinocembrin	Rosemary, Sage; Dill; Jujube, Longan And Chaste; Acacia, Sunflower, Linden, Basil, Citrus, Buckwheat, Goldenrod, Black Locust, Sulla, Thistle	MeOH:water (1:1); MeCN	CE–ESI-MS; UHPLC-DAD MS/MS	[65,69,70,74]
Pinostrobin	Sage	MeCN	UHPLC-DAD MS/MS	[65]
Flavones				
Chrysin	Rosemary; Kelulut, Tualang; Buckwheat, Manuka; Sulla, Thistle, Black locust, Citrus; Lavender, Eucalyptus, Thyme, Chestnut; Sage; Sunflower, Linden, Basil, Buckwheat; *Ziziphus spina-christi*	MeOH/water (1:1); MeOH; MeCN; 40/50/10 (*v*/*v*/*v*) water/TFA/MeCN	CE–ESI-MS; LC-ESI-MS/MS; UHPLC–UV; HPLC-DAD-TOF-MS	[65,67,69,71,72,76,80]
Acacetin	Acacia	MeOH	HPLV-UV	[77]
Luteolin	Black locust, Sulla, Thistle, Citrus*,* Manuka, Tualang, Sunflower; Rhododendron, Rosemary; Raspberry, Orange, Cherry Blossom, Dandelion, Melon, Lavender, Sage, Rapeseed, Sunflower, Linden, Basil, Buckwheat, Thyme, Pine, Sage	MeOH; MeOH/water (20:80,*v*:*v*)	UHPLC–UV; HPLC-CEAD HPLC-ESI-MS	[72,80,81]
Baicalein	Lavender, Orange Blossom, Rosemary, Heather, Eucalyptus, Chestnut and Thyme	MeCN	HPLC-DAD-TOF-MS	[78]
Flavanones				
Hesperetin	Citrus; Lavender, Orange Blossom, Rosemary, Heather, Eucalyptus, Chestnut;Thyme; Sage, Sulla, Thistle, Rhododendron; Phacelia, Pumpkin, Raspberry	MeOH; MeCN	HPLC-ECD; HPLC-DAD-TOF-MS; UHPLC-DAD MS/MS	[65,78,81,82]
Naringenin	Lavender, Orange Blossom, Rosemary, Heather, Eucalyptus, Chestnut and Thyme; *Ziziphus Spina-Christi*	MeCN	HPLC-DAD-TOF-MS	[78,83]
Eriodictyol	Sunflower	MeCN	UHPLC-HESI-MS	[72]
Dihydroflavonols				
5-methoxy pinobanksin	Acacia, Black locust	MeOH	HPLC-PDA	[77]
Phenolic acids				
*P*-Hydroxybenzoic acid	Acacia, Clover, Heather, Manuka, Buckwheat; Wild Chrysanthemum, Milk Vetch, Jujube, Sage; Sulla, Dill; Black locust, Rapeseed, Lime, Goldenrod, Heather, Buckwheat; Cornflower	MeOH MeCN	HPLV-UV; HPLC-DAD; HPLC–ECD-DAD	[65,71,77,84,85,86]
Vanillic acid	Black locust, Heather, Liven vine; Christmas vine; Rapeseed, Lime, Heather, Cornflower, Buckwheat, Black Locust	MeOH	HPLC-DAD-ESI-MS/MS; HPLC–ECD-DAD	[66,77,85,86]
Phenylacetic acid	Sweet chestnut; Sage; Chestnut, Eucalyptus, Sulla; Black Locust, Lime, Lavender, Rapeseed, Sunflower, Rosemary, Orange, Lemon	MeOH	GC–MS; HPLC-UV	[79,87]
L-β-phenyllactic acid	Chestnut, Eucalyptus, Sulla; Black Locust, Lime, Lavender, Rapeseed, Sunflower, Rosemary, Orange, Lemon	MeOH	HPLC-UV	[87]
Dl-*p*-hydroxy-phenyllactic acid	Chestnut, Eucalyptus, Sulla; Black Locust, Lime, Lavender, Rapeseed, Sunflower, Rosemary, Orange, Lemon	MeOH	HPLC-UV	[88]
Gentisic acid	Sage	MeCN	UHPLC-DAD MS/MS	[65]
Rosmarinic acid	Sage, Rapeseed, Lime, Heather, Cornflower, Buckwheat and Black Locust	MeCN; MeOH	UHPLC-DAD MS/MS; HPLC–ECD-DAD	[65,85,86]
Phenyllactic acid	Heather, Thistle, Manuka, Cornflower	MeCN	HPLC–DAD	[79]
Lumichrome	Cornflower	MeCN	HPLC–DAD	[79]
Hydroxycinnamic acid				
Caffeic acid	Black locust, Liven vine; Christmas vine, Buckweat, Manuka, Citrus; Acacia, Milk vetch, Wild Chrysanthemum, Jujube flower; Chestnut, Eucalyptus, Sulla; Coriandrum, Gelam, Pine, Rapeseed, Lime, Heather, Cornflower, Buckwheat And Black locust	MeOH/water 1:1; MeOH 43%:HCOOH (57%, *v*/*v*)	HPLC-DAD-ESI-MS/MS; HPLC-ECD; HPLC-DAD; HPLC-UV	[66,68,82,84,85,86]
Caffeic acid phenethyl ester	Kelulut	MeOH/water 1:1	LC-ESI-MS/MS	[67]
*P*-Coumaric acid	Liven vine; Christmas vine; Tualang, kelulut; Citrus; Acacia, Milk vetch, Wild chrysanthemum, Jujube flower; Chestnut, Eucalyptus, Citrus, Sulla, Mint, Thymus	MeOH; MeOH/water 1:1; MeOH 43%:HCOOH (57%, *v*/*v*)	HPLC-DAD-ESI-MS/MS; HPLC-ECD; HPLC-DAD; HPLC-UV	[66,68,82,84]
*O-*coumaric acid	Chestnut, Eucalyptus, Sulla; Black locust, Lime, Lavender, Rapeseed, Sunflower, Rosemary, Orange, Lemon	MeOH	HPLC-UV	[87]
*M*-coumaric acid	Chestnut, Eucalyptus, Sulla; Black Locust, Lime, Lavender, Rapeseed, Sunflower, Rosemary, Orange, Lemon	MeOH	HPLC-UV	[87]
Ferulic acid	Liven vine; Christmas vine; Citrus; Acacia, Milk vetch, Wild Chrysanthemum, Jujube flower; Black locust, Buckweat; Chestnut, Eucalyptus, CitrusSulla; *Sesamum indicum*	MeOH; MeOH/water 1:1; MeCN	HPLC-DAD-ESI-MS/MS; LC-ESI-MS/MS; HPLC-ECD; HPLC-PDA; LC-DAD; HPLC-DAD	[64,66,67,68,77,79,82,85]
Cinnamic acid	Black locust, Tualang, kelulut; Rapeseed, Lime, Heather, Cornflower, Buckwheat, Black locust	MeOH/water 1:1	LC-ESI-MS/MS; HPLC-PDA; HPLC–ECD-DAD	[67,85,86]
Trans-cinnamic acid	Chestnut, Eucalyptus, Sulla; Acacia, Lime, Lavender, Rapeseed, Sunflower, Rosemary, Orange, Lemon	MeOH	HPLC-UV	[87]
2-Hydroxycinamic acid	Tualang, Kelulut	MeOH/water 1:1	LC-ESI-MS/MS	[67]
3,4-dimethoxycinnamic acid	Black locust	MeOH	HPLC-PDA	[77]
T-cinnamic acid	Black locust	MeOH	LC-MS;	[77,88]
Isoferulic acid	Black locust	MeOH	HPLC-PDA	[77]
Sinapic acid	Rapeseed, Lime, Heather, Cornflower, Buckwheat and Black locust	MeOH	HPLC–ECD-DAD	[85,86]
Hydroxybenzoic acids				
Syringic acid	Linen vine, Kelulut, Tualang; Kanuka, Acacia, Milk vetch, Wild Chrysanthemum, Jujube flower, Sulla, Thistle, Citrus	Metanol; MeOH/water 1:1; MeOH/water 1:1; MeOH 43%:HCOOH (57%, *v*/*v*)	HPLC-DAD-ESI-MS/MS; LC-ESI-MS/MS; HPLC-DAD; UHPLC–UV	[66,67,80,84,89]
Gallic acid	Tualang, kelulut; *Ziziphus Spina-Christi*, Acacia, *Prosopis juliflora* (mesquite); Acacia, Chestnut, Savory, Sulla, Ailanthus, Thymus, Orange, Cornlfower, Rapeseed, Citrus, Heather, Eucalyptus	MeOH/water 1:1; MeOH 43%:HCOOH (57%, *v*/*v*); MeCN	LC-ESI-MS/MS; HPLC-DAD; HPLC-UV	[67,76,84]
Vanillic acid	Black locust, Sulla, Thistle, *citrus*, *Prosopis juliflora* (mesquite), Manuka, *Ziziphus Spina-Christi;* Lavender, Rosemary, Sulla, Rapeseed, Lime, Heather, Cornflower, Buckwheat, Thistle	MeOH	UHPLC–UV	[81,85,86,87]
Ellagic acid	Rapeseed, Lime, Heather, Cornflower, Buckwheat, Black locust	MeOH	HPLC–ECD-DAD	[85,86]
Benzoic acid	Black locust, Buckweat, Manuka; Chestnut, Eucalyptus, Sulla; Acacia, Lime, Lavender, Rapeseed, Lavender, Sunflower, Rosemary, Orange, Lemon	MeOH	HPLV-UV; HPLC-PDA	[68,77,87]
*P*-hydroxybenzoic acid	Acacia, Buckwheat, Cornflower, Milk Vetch, Dill, Citrus, wild chrysanthemum, Jujube Flower; Sage, Sulla	MeOH/water 1:1; MeOH 43%:HCOOH (57%, *v*/*v*); MeCN	HPLC-DAD; UHPLC-DAD MS/MS	[65,70,84]
3-Hydroxybenzoic acid	Buckwheat; Chestnut, Eucalyptus, Sulla; Acacia, Lime, Lavender, Rapeseed, Sunflower, Rosemary, Orange, Lemon	MeOH	LC-DAD	[79,87]
4-Hydroxybenzoic acid	Kelulut, *Paliurus spina-christi* Mill.; Chestnut, Eucalyptus, Sulla; Acacia, Lime, Lavender, Rapeseed, Sunflower, Rosemary, Orange, Lemon	MeOH/water 1:1; MeOH	LC-ESI-MS/MS; GC–MS	[67,79,87]
4-methoxybenzoic acid	*Paliurus spina-christi* Mill.; Heather; Manuka, Kanuka	MeOH	GC–MS; HPLC-MS/MS	[79,89]
Dihydroxybenzoic acids				
Protocatechuic acid	Acacia, Buckweat, Cornflower, Manuka, Heather, Pine; Milk Vetch, Wild Chrysanthemum, Jujube Flower; Chestnut, Eucalyptus, Lavender, Rapeseed, Sunflower, Rosemary, Orange, Lemon, Black Locust, Sulla, *Echium plantagineum*	MeOH 43% (*v*/*v*) and HCOOH (aq), pH 2.54 (57%, *v*/*v*)	HPLV/UV; HPLC-DAD	[71,75,84,87]
Benzoic acids derivatives				
Methyl syringate	Asphodel, Manuka, Kanuka, Sulla, Dill, Lemon, Orange, And Medlar	MeCN 2 Water:MeCN 60:40 (*v*/*v*)	HPLC-DAD; HPLC-MS/MS	[70,79]
Tanins				
Monogalloyl-glucose	Rosemary	MeOH:water (1:1)	CE–ESI-MS	[69]
		Monoterpenoids		
Carvacrol	*Ziziphus Spina Christi*	MeOH	HPLC-DAD	[90]
Thymol	*Ziziphus Spina Christi*	MeOH	HPLC-DAD	[90]
Other polyphenols				
Chlorogenic acid	Buckweat, Manuka; Acacia, Milk Vetch, Wild Chrysanthemum, Jujube Flower; Acacia, Chestnut, Savory, Sulla, Ailanthus, Thymus, Orange	MeOH; MeOH 43% (*v*/*v*) and HCOOH (aq), pH 2.54 (57%, *v*/*v*); 40/50/10 (*v*/*v*/*v*) water/TFA/MeCN	HPLV-UV	[68,71,76,84]
Gallocatechin	Sweet chestnut, Eucalyptus, Citrus, Sulla, Sage	MeOH; MeCN	HPLC-MS/MS; UHPLC-DAD MS/MS	[65,89]
Epigallocatechin	Sage	MeCN	UHPLC-DAD MS/MS	[65]
Epigallocatechin gallate	Sage	MeCN	UHPLC-DAD MS/MS	[65]
Resveratrol	Sage	MeCN	UHPLC-DAD MS/MS	[65]
Other compounds				
Phenyllactic acid	Cornflower, Manuka, Kanuka, Thistle, Mint, Heather, Sulla, Dill	MeOH	HPLC-MS/MS	[70,85,86,89]
2-cis,4-trans-abscisic acid	Strawberry tree, manuka, Black Locust, Buckwheat, Basil, Goldenrod, Linden, Sunflower, Rapeseed	MeCN Water/MeCN 60:40 (*v*/*v*)	HPLC-DAD HPLC-MS/MS	[79]
2-trans,4-trans-abscisic acid	Strawberry tree, Manuka, Cornflower	MeCN Water/MeCN 60:40 (*v*/*v*)	HPLC-DAD HPLC-MS/MS	[79,91]
Fisetin	Lavender, Orange Blossom, Rosemary, Heather, Eucalyptus, Chestnut, Thyme	MeCN	HPLC-DAD-TOF-MS	[78]

UHPLC, Ultra-High-Performance Liquid Chromatography; LC-MS, Liquid chromatography-mass spectrometry; MeCN, acetonitrile; MeOH, methanol; TFA,trifluoroacetic acid.

**Table 3:** Physicochemical properties of monofloral honeys from different geographical origin.

Honey Type	Origin	Moisture Content (%)	Acidity (meq/kg)	Sugar Composition (%)	Ash (%)	HMF (mg/kg)	Reference
*Allium ursinum* L.	Hungary	18.3–19.7	19.0–23.5	n.i.	n.i.	15.3–20.6	[98,99]
*Arbutus unedo*	Portugal	15.8–19.8	20.17–61.10	Fructose: 35.96 Glucose: 26.743	0.70	8.2	[45,100]
*Asclepias* sp.	Hungary	16.9–20.0	29.0–34.0	Fructose: 41.1 Glucose:30.8	n.i.	20.3–25.0	[98,99]
*B. napus*	Romania	18.4	16	Fructose: 35.26 Glucose: 31.78 F/G ratio:1.11	n.i.	13.3	[36]
	Bulgaria	n.i.	n.i.	Fructose: 36.70 Glucose: 37.46	n.i.	40.65	[101]
	Hungary	17.2–19.8	21.5–27.5	Fructose: 38.5 Glucose:40.5	n.i.	0.8–17.9	[98,99]
*Calluna vulgaris* L.	Portugal	19.0	35.23	Fructose: 35.963 Glucose: 26.743	0.48	22.80	[100]
	Turkey	20.86	n.i.	Fructose: 45.11 Glucose: 25.00 F/G ratio: 1.80	n.i.	62.24	[25]
*Carlina racemosa*	Portugal	17.2	41.37	Fructose: 35.703 Glucose: 25.020	0.55	n.i.	[100]
*Castanea sativa* Mill.	Hungary	17.0–17.4	26.0–32.0	n.d.	n.d.	12.6–33.1	[98,99]
	Turkey	19.70	n.i.	Fructose: 38.44 Glucose: 19.35 F/G ratio: 1.98	n.i.	9.28	[25]
*Ceratonia siliqua*	Portugal	15.40–18.6	31.72–56.20	Fructose: 37.964 Glucose: 26.372	0.43	41.80	[45,100]
*Cistus sp.*	Portugal	15.90	32.0	n.i.	0.25	20.0	[102]
*Citrus sinensis*	Portugal	15.8–18.2	19.40–30.50	Fructose: 40.180 Glucose: 27.110	0.13	28.20	[45,100]
*Coriandrum sp.*	Hungary	19.1–19.3	40.2–43.5	n.i.	n.i.	8.2–14.5	[98,99]
	Bulgaria	16.27	4.71–16.09	Fructose: 40.25 Glucose: 26.11	n.i.	17.38–22.72	[101,103]
*Cytisus scoparius*	Portugal	19.70	30.0	n.i.	0.37	6.56	[46]
*Echium sp.*	Portugal	16.80	25.0	n.i.	0.23	94.0	[102]
*Erica sp.*	Portugal	17.31–20.60	15.5–47.70	Fructose:34.4–37.1 Glucose:28.0–33.4	0.32–0.36	4.63–20.40	[45,46,104]
	Spain	18.19	35.66	n.d.	0.47	3.72	[105]
*Eucaliptus* sp.	Portugal	14.30–19.20	12.6–29.7	Fructose: 23.34 Glucose: 35.82	0.07–0.46	2.54–32.75	[46,100,105]
*H. annus*	Romania	16.23–20.39	15–94–47.32	Fructose: 36.74 Glucose: 28.37 F/G ratio: 1.33	0.33–0.36	2.66–10.96	[106,107]
	Bulgaria	n.i.	n.i.	Fructose: 40.91 Glucose: 35.80	n.i.	25.47	[101]
	Portugal	19.2	25.50	Fructose:41.24 Glucose:38.09	0.15	8.10	[100]
	Hungary	17.2–19.7	33.5–46.5	Fructose: 40.5 Glucose:36.5	n.i.	0.6–25.2	[98,99]
Honeydew honey	Hungary	19.9–20.0	42.0–48.5	Fructose: 37.3 Glucose:27.6	n.i.	19.6–34.2	[98,99]
*Lavandula stoechas* L.	Hungary	19.0–19.1	29.5–35.5	n.i.	n.i.	1.0–13.0	[98,99]
	Portugal	13.56–19.20	16.40–35.10	Fructose:39.81–-41.66 Glucose: 26.40–28.52	0.09–0.18	5.35–12.80	[45,46,100]
	Turkey	17.15	n.i.	Fructose: 32.65 Glucose: 22.19 F/G ratio: 1.47	n.i.	24.42	[25]
*Manuka*	New Zealand, Australia, Malaysia	11.59–20.27%	42.67 ± 3.01	Fructose: 33.0–40.0 Glucose: 27.8–36.2	0.21 ± 0.01	31.53–40.0	[19,71]
*Tualang*	Malaysia	16.39–22.32	44.92–86.08	Fructose: 29.60–41.73 Glucose: 30.00–47.13 F/G ratio: 0.88		5.08–1226.32	[19,108]
*Gelam*	Malaysia	18.51–20.33	46.50–59.75	Fructose: 44.90 Glucose: 50.44 F/G ratio: 0.89		8.52–10.20	[19,108]
*Longan*	Thailand	20.11	17.60	Fructose: 41.02 Glucose: 34.91 F/G ratio: 1.18	0.23	0.58	[109]
*Mentha pulegium*	Portugal	18.10	42.17	Fructose: 36.41 Glucose:24.66	0.61	4.10	[100]
*Mentha x piperita*	Romania	17.7	26.9	Fructose: 36.03 Glucose: 27.87 F/G ratio: 1.30	0,15	29.2	[36]
*Pinus sp.*	Turkey	15.5–15.8	26.5- 28.6	Fructose: 32.1–39.8 Glucose: 23.67–28.2 F/G ratio: 1.17–1.68	0.50–0.55	3.57	[25,110,111,112,113]
*Pinus sp*	Greece	n.i.	n.i.	Fructose: 10.33 Glucose: 2.52 F/G ratio: 4	n.i.	12.34	[114]
*R. pseudoacacia*	Bulgaria	n.i.	n.i.	Fructose: 43.22–42.76 Glucose: 27.50–24.13	n.i.	17.82	[102,115]
	Hungary	17.1–19.9	13.0–27.5	Fructose: 43.6 Glucose: 29.1	n.i.	1.1–29.4	[98,99]
*Rhododendron sp*	Italy	n.d.	28.00	Fructose: 27.80 Glucose: 38.20	n.i.	3.40	[40]
	Turkey	18.89	34.33	Fructose: 43.58 Glucose: 23.16 F/G ratio: 1.88	n.i.	3.20	[25,116]
*Rubus sp.*	Romania	18.3	27.3	Fructose: 36.30–33.46 Glucose: 38.15–29.00 F/G ratio: 1.04–1.26	0.340	18.7	[36,117]
*Thymus sp.*	Romania	17.3	22.5	Fructose: 36.77 Glucose: 26.86 F/G ratio: 1.38	n.i.	30.8	[36]
	Italy	n.d.	38.50	Fructose: 37.80 Glucose: 30.50	n.i.	30.40	[16]
	Greece	n.i.	n.i.	Fructose: 11.51 Glucose: 5.15 F/G ratio: 2.2	n.i.	203	[114]
*Thymus vulgaris*	Portugal	15.80–17.50	37.20–69.50	Fructose:37.04 Glucose:24.79	0.45	1.60	[45,100]
*Tilia sp.*	Bulgaria	n.i.	n.i.	Fructose: 40.13 Glucose: 27.07	n.i.	n.i.	[101]
	Italy	n.i.	12.80	Fructose: 37.20 Glucose: 30.20	n.i.	3.50	[16]
	Hungary	17.6–19.9	21.0–41.5	Fructose: 40.0 Glucose: 30.6	n.i.	4.3–37.5	[89,99]
*Ziziphus sp.*	Egypt	15.1–20.20	12.5–29.17	Fructose: 39.5–42.1 Glucose: 29.5–32.0 F/G ratio: 1.30–1.36	0.178	0.6–1.6	[118]
*Hovenia dulcis*	South Korea	17.0–19.73	8.50–33.50	Fructose: 33.1–44.5 Glucose: 20.2–29.3 F/G ratio: 1.27–2.23	0.08–0.82	0.00	[119,120]

**Table 4:** Monofloral honeys mineral content from different countries.

Honey Type	Origin	K (mg/kg)	Mg (mg/kg)	Ca (mg/kg)	Na (mg/kg)	References
*Acacia mangium*	Malaysia	1459.33–413.63	44.97–21.83	119.80–567.27	458.95–180.23	[144]
*Arbutus unedo*	Portugal	1736.29	24.92	50.00	161.02	[100]
*Asclepias* sp.	Hungary	262–342	6.81–8.88	17.8–24.1	4.51–5.92	[48]
*Brassica* sp.	Romania	194.17–112.56	23.90–23.47	88.63–87.14	47.96–36.08	[145]
	Bulgaria	105	11	46	8.49	[146]
	Hungary	162–292	11.2–16.9	33.4–50.6	6.13–9.09	[48]
	Hungary	160–280	11–16	36–48	5.6–12	[47]
*C. sativa*	Bulgaria	16.28	16	66	9.55	[146]
	Spain	221.2–269.4	107.89–962.64	122.16–111.42	22.30–26.20	[147]
	Italy	290.00–5300	45.0–201.0	23.00–352.0	64.0–104.0	[148]
	Hungary	1563–2186	30.1–41.2	81.9–116	10.8–14.6	[48]
*Calluna vulgaris*	Portugal	1196.31	31.51	45.15	155.45	[100]
*Carlina racemosa*	Portugal	1341.16	46.63	42.12	208.10	[100]
*Ceratonia siliqua*	Portugal	723.28	46.89	79.09	138.10	[100]
*Citrus limon* L.	Italy	186–3110	59.00–152	36.00–289	41.00–118.0	[148]
*Citrus sinensis*	Portugal	170.07	9.81	28.18	56.34	[100]
	Spain	735.21	54.15	43.10	12.04	[146]
*Citrus sp.* Orange blossom	Italy	168.00–3016.0	84.00–202.00	177.00–318.00	39.00–145.00	[148]
*Coriandrum sativum*	Bulgaria	564	7.1	44	14.20	[149]
*Cytisus scoparius*	Portugal	160.06	31.97	71.41	101.22	[46]
*Erica sp.*	Portugal	166.70	36.78	35.14	174.45	[46]
*Eucalyptus sp.*	Italy	112.0–372.0	42.00–331.0	84.0–232.0	293.0–928.0	[148]
	Portugal	397.17–2040.50	25.04–48.84	19.90–122.45	151.62–667.39	[46,100]
*Forest honey*	Hungary	1331–2212	39.7–60.2	52.4–75.5	10.8–17.9	[48]
Gelam	Malaysia	1363.40	31.63	275.77	196.84	[19,109]
*H. annus*	Romania	234.64–1111.1	24.9–30.3	67.7–97.4	13.2–36.4	[145]
	Bulgaria	280–247	14	71	7.58	[33,149]
	Portugal	276.86	24.92	68.18	87.93	[100]
	Hungary	502–735	21.8–33.3	82.9–124	6.37–9.32	[48]
*Hedysarum coronarium* L	Italy	227.0–295.0	92.0–134.0	188.0–291.0	519.0–681.30	[148]
*Lavandula stoechas*	Portugal	78.09–173.17	6.84–14.46	13.38–32.43	41.47–95.02	[46,100]
*Dimocarpus longan* Lour.	Malaysia	906.35	35.47	118.07	95.94	[144]
*Mentha pulegium*	Portugal	158.256	70.92	39.09	161.64	[100]
*Pauliurus spina christi*	Bulgaria	1198	17	62	11.80	[146]
*Persea americana* Mill.	Spain	557.073	623.58	55.97	69.05	[141]
*Phacelia tanacetifolia*	Hungary	102–130	4.09–5.16	9.12–12.5	3.02–3.81	[48]
*Ananas comosus* (L.) Merr.	Malaysia	473.68	36.63	74.60	111.29	[144]
*Pinus sp.*	Turkey	1832–1989	54.2- 59.2	50.1- 59.9	n.i.	[110,111,113]
*R. pseudoacacia*	Italy	400–1150	41.0–93.0	55.0–182.0	60.0–1190	[148]
	Romania	244.58–146.66	6.72–3.25	6.94–1.02	24.32–8.32	[150,151]
	Bulgaria	250–126	6.0	32.0	8.11	[34,145,149]
	Hungary	115–176	3.83–5.30	10.2–15.5	3.13–4.62	[48]
*Rosmarinus officinalis* L (Rosemary)	Spain	253.00–553.03	9.80–42.11	15.14–206.70	9.18–36.80	[141,147]
*Thymus vulgaris* L	Portugal	341.91	74.15	68.79	61.30	[100]
	Spain	322.45–1502.00	40.70–341.74	98.10–181.69	36.0–151.65-	[147]
*Tilia* sp.	Bulgaria	112.3–796	21	77	7.50	[34,146]
	Hungary	921–1280	17.1–24.9	71.8–98.3	10.1–13.9	[48]
*Kompassia excelsa*	Malaysia	1576.40	35.03	165.10	268.23	[144]
*Vicia sp.*	Bulgaria	196	10	33	9.62	[146]
Wildflower	Italy	270–2460	85.0–184.0	168.0–387.0	322.8–1321.4	[148]
*Ziziphus* sp.	Egypt, Palestina	1569.3–476.40	34.48–22.1	136.6–94.56	115.04–49.2	[118]
*Hovenia dulcis*	South Korea	405.3–409.7 mg/L	9.9–11.5	19.3–20.8	1.6–2.0	[120,150,152]

**Table 5:** Phenolic compounds, antioxidant capacity, and in vitro pharmacological studies of monofloral honeys.

Honey Type	Origin	TPC (mg GAE/100 g)	TFC (mg QE/100 g)	Antioxidant Activity	In Vitro Pharmacological Activity	Reference
Acacia Honey (*Acacia* sp.)	Burkina Fasan, Pakistan, Malaysia	93.43–14.70 mg GAE/100 g	6.14–1.13 mg QE/100 g	DPPH, IC_50_ (mg/mL): between 10.40–17.97 ABTS (176.66–231.5 μmol TE/g) ORAC (30.62–83.72 μmol TE/g)	↓ IL 1β level and ↓ TNF-α level after 24 h with increase in honey concentration 1–8% (*v/v*) ↑ calcium ion level from 24 to 48 hrs incubation periods ↑ anticancer activity on PC-3 cell line after 24 hrs incubation with IC_50_: 4.43% (*v/v*) ↓ Cell viability and ↑ Apoptotic cell death in MCF-7 breast cancer cells at 3.12–100% (*v/v*) for 24–72 hrs ↓ TNF-α, IL-1β, Ca ion in NCI-H460 non-small lung cancer cells	[154,155,156,157,158,159,160,161,162,163,164,165,166,167]
Astragalus honey (*Astragalus microcephalus* Willd)	Turkey, Iran	198.00 CE/100 g	23.57 mg QE /100 g	DPPH: 7.2 mg/mL	↓ in Bcl-2 mRNA expression ↓ Bcl-2 gene in 5637cells ↓ p53 gene in HepG2 cells ↓ expression of the p53 gene by 80% in 5637 cells ↔ to ↓ p53 gene expressions in L929 cells	[158]
Berry honey *Rubus sp.*	Romania	19.9 mg GAE/100 g	33.5 mg QE/100 g	DPPH: 79.05%	n.i.	[36]
Black locust honey (*Robinia pseudoacacia*)	Malaysia, Poland, Turkey, Romania	2.0–39.0 mg GAE/100 g	0.91–2.42 mg QE/100 g	DPPH: 12.72–29.98 mg GAE/100 g honey; FRAP: 82.39 mg TE/100 g	↓ LPO in liver ↓ Cell viability in B16-F1 cells after 24 hrs at doses of 0.2 and 0.1 g/mL ↓ Cell viability in A375 line observed after 48- and 72-h exposure of cells to 0.2 and 0.1 g/mL doses of acacia honey ↓ Cell viability and ↓ Bcl-2, p53 in NCI-H460 non-small lung cancer cells at 0.5–8% (*w*/*v*) for 48 hrs Arrest cell cycle at G0/G1 phase in B16-F1 and A375 line	[18,24,159,160,161]
	Italy	112.99 mg GAE/kg	67.32 mg QE/kg	DPPH, IC_50_ (mg/mL): 21.56 FRAP, (mmol Fe(II)/Kg honey): 1.377	n.i.	[76]
	Poland	142.8 mg GAE/kg	n.a.	DPPH, (mmol TEAC/kg): 0.3 FRAP, (mmol Fe(II)/Kg honey): 0.6	n.i.	[87]
Buckwheat honey *(Fagopyrum esculentum)*	Poland	1113.0 mg GAE/100 g	n.a.	DPPH, (mmol TEAC/kg): 1.2 FRAP, (mmol Fe(II)/Kg honey): 5.7	↑ ROS inhibition produced by human PMNs (160 to 130 mL/g activity) ↑ Superoxide anion scavenging inhibition (RIC50: 290 mL/g) ↓ oxidative stress, ↓ LPO in liver	[87,162]
Canola (*B. napus*)	Poland	47.71−183.0 mg GAE/100 g	0.72 mg CEQ/100 g	DPPH: 55.4% DPPH: 18.22 μmol TE/ 100 g FRAP: 92.05 μmol TE/100 g TEAC: 67 μmol TE/100 g	↓ Superoxide radical, ↓ LPO	[47,87,163]
	Romania	23.7–19.9 mg GAE/100 g	20.2–2.5 mg QE/100 g	n.i.	n.i.	[36,164]
Chestnut honey (*Castanea sativa*)	Italy	14.26–94.56 mg GAE/100 g	12.52–143.63 mg QE/100 g	DPPH, I% (%): 75.37	↓ Oxidative stress ↑ inhibition of LPS-induced NO ↑ antimutagenic activity on TA98 strain (10 µg/mL to 20 mg/mL honey concentration) ↑ Apoptotis in MCF-7 cell line after 48 h at 2.5 and 5 mg/mL doses ↑ Apoptotis in SKBR-3 cell line at 5 mg/mL dose of chestnut honey (62.05% cell death) ↔ apoptosis at 5 mg/mL dose on MDA-MB-231 (25.87% cell death) ↔ apoptosis at 5 mg/mL dose of the chestnut honey on MCF-10A (31.5% cell death)	[44,45,62,68,76,165,166,167,168]
	Turkey	5.49–8.01 mg GAE/100 g	0.99–2.49 mg QE/100 g	DPPH: 17.66–20.05 mg/mL FRAP: 2.056–4.30 mmol Fe(II)/Kg honey	↓ MPO, ulcer index, microvascular permeability	[25]
Orange honey (*Citrus sinensis*)	Portugal	32.10 mg GAE/100 g	1.73 mg QE/100 g	n.i.	n.i.	[100]
	Italy	84.37 −32.10 mg GAE/100 g	69.8 mg QE/100 g 9	DPPH, IC_50_ (mg/mL): 25.87 FRAP, (mmol Fe(II)/Kg honey): 1.265 TEAC:4.04 mg/mL ORAC: 3.28 mmol TE/g	↓ Oxidative stress ↑ antibacterial activity	[76]
*Citrus sp.* *honey*	Italy, Egypt	12.08 mg GAE/100 g	5.82 mg QE/100 g	DPPH, I% (%): 55.06	↑ inhibitory activity in MCF-7 (52.53%) ↔ inhibitory activity in Caco-2 (35.68%) and Hep-G2 (32.47%)	[68,168]
Clover honey (*Trifolium pratense L., T repens L.*)	Croatia	100.4 mg GAE/100 g	3.9 mg QE/100 g	DPPH: 23.3% FRAP: 89.0 µM Fe(II)	↓ LPO, inhibits activity of NO, TNF-α and IL-6 ↓ AST, ALP ↔ inhibitory activity in Caco-2 (30.94–49.84%) ↔ inhibitory activity in MCF-7 (15.45–28.14%) ↓ inhibitory activity in Hep-G2 (1.75–24.84%)	[168,169,170]
Coriander honey (*Coriandrum* sp.)	Bulgaria	68.70 mg GAE/100 g	8.02 mg QE/100 g	FRAP: 380.66(μmol Fe [II]/100 g) DPPH: 29.94%	↓ LPO, SOD, ↑ GSH level in liver	[171]
Cornflower honey (*Centaurea cyanus*)	Poland	44.06 mg GAE/100 g	n.i.	DPPH IC_50_: 44.40 mg/mL FRAP IC_50_: 3.85 mg/mL	↑ antibacterial activity ↑ wound healing	[86]
Cotton honey *(Gossypium* sp.)	Egypt	45.42 mg GAE/100 g		DPPH (SC_50_ (µg/mL): 99.40 ABTS^+^ SC_50_ (µg/mL): 41.20	↔ inhibitory activity in MCF-7 (32.91%) ↓ inhibitory activity in Caco-2 (29.25%) and Hep-G2 (20.08%)	[168]
Eucalyptus honey (*Eucalyptus* sp.)	Portugal	54.25 mg GAE/100 g	5.28 mg QE/100 g	TEAC:2.86 ORAC: 7.40	n.i.	[51]
	Australia	106.7 mg GAE/100 g	3.6 mg QE/100 g	DPPH:44.3 μmol TE/100 g FRAP:142.97 μmol TE/100 g	↓ Superoxide radical, ↓ LPO	[169,172,173]
	Italy	11.08 mg GAE/100 g	6.16 mg QE/100 g	DPPH, I% (%): 73.04	↑ inhibition activity in MCF-7 cell line (159.4 ± 3.6 μg/mL)	[68,174]
European goldenrod or woundwort *Solidago virgaurea* L)	Poland	11.29–21.03 mg GAE/100 g	0.93–1.41 mg QE/100 g	DPPH: 31.08−39.46% ABTS: 46.69–56–92%	n.i.	[174]
Fennel honey *(Foeniculum vulgare)*	Egypt	29.1–102.0 mg GAE/100 g	17.7− 27.0 mg QE/100 g	Cellular antioxidant activity: 5.66–26.4 µmol of QE/100 g	↔ inhibitory activity in MCF-7 (45.93%) ↓ inhibitory activity in Caco-2 (25.53%) and Hep-G2 (11.75%)	[168,175]
Gelam honey *(Kompassia eroxi)*	Malaysia	74.12 mg GAE/100 g	46.11 mg QE/100 g	DPPH IC_50_ (6.68 ± 0.28) mg/mL FRAP: 115.61 ± 3.86 μmol Fe [II]/100 g	↑ Antioxidant enzyme activities ↓ Hypertriglyceridemia and pro-oxidative effects ↑ apoptosis in HepG2 cells at 3% concentration after 24 h ↑ apoptosis in WRL-68 cells at 6% after 24 h ↓ MDA levels in HIT-T15 cells ↑ insulin content	[176,177]
Goldenrod honey (*Solidago sp.*)	Poland	173.4 mg GAE/100 g	n.a.	DPPH: (mmol TEAC/kg): 0.2 FRAP: (mmol Fe(II)/Kg honey): 1.0	n.i.	[87]
Heather honey (*Calluna vulgaris*)	Portugal	117.59 mg GAE/100 g	21.16 mg QE/100 g	TEAC:0.86 ORAC: 22.58	↑ apoptosis in HL-60 cells with 50 mg/mL concentration of heather honey for 48 h (70.4–78.5%) ↑ antiproliferative activity 72 h treatment with 100–250 mg/mL of heather honey (11.9–7.1% of survival) ↓ ROS levels after 24 h ↓ DNA strand breaks (0.1 mg mL^−1^, 15%) induced by BaP in HepG2 cells	[100,178,179]
	Poland, Portugal	306.2−269.03 mg GAE/kg	n.a.	DPPH IC_50_: (24.6 (SD 0.2) mg/mL, FRAP (1948 mg/kg TEAC/kg): 0.6 FRAP: (mmol Fe(II)/Kg honey): 2.1	Protection of HepG2 against mutagens-induced DNA damage	[87]
Lavander honey (*Lavandula sp.*)	Portugal	31.85–34.13 mg GAE/100 g	3.09–3.15 mg QE/100 g	DPPH IC_50_: 5.3 mg/mL TEAC: 3.98–4.03 ORAC: 7.43–7.57	Diabetic foot ulcers healing activity through reducing ROS	[44,100]
Linden honey (*Tilia sp.*)	Romania, Slovenia, Poland	16.0–85.8 mg GAE/kg 192.5 mg GAE/100 g	4.70–6.98 mg QE/100 g	DPPH IC_50_: 42.77 mg/mL, FRAP: 137.8 mg/kg DPPH: (mmol TEAC/kg): 0.4 FRAP: (mmol Fe(II)/Kg honey): 1.4	↓ Oxidative stress and ROS	[27,87,180]
Manuka honey *Leptospermum scoparium J*.R. et G.Forst	New Zealand	1288.0 GAE µg/g	37.64 CE µg/g	DPPH: 18.69 µmol TE/g FRAP: 3.68 µmol TE/g; TEAC: 30.72 µmol TE/g	↑ GPx, ↓ CAT activity, ↓ DNA damage and MDA level, protects proteins and lipids against AAHP-induced stress	[159,181,182]
		429.61 mg GAE /kg	97.62 CE mg/kg	DPPH: 0.06 mmol TE/ 100 g FRAP: 0.14 mmol TE/ 100 g TEAC: 0.22 mmol TE/100 g	↓ viability of B16 F1 cells (0.3% manuka) after 24 h ↑ apoptosis in CT26 and MCF-7 cells with 2.5% manuka	[129,130,182]
Oak honey (*Quercus sp.*)	Turkey	115.41 mg GAE/100 g	77.36 mg QE/100 g	Inhibition of ABTS %: 89.36	50% inhibition after 24 h incubation of AGS cells with the 1.7% final concentration ↓ ROS generation at the concentration of 0.25% (*w/v*) in AGS cells DNA damage	[183]
Pine honey (*Pinus sp.*)	Greece, Turkey	61.42–163.98 mg GAE/100 g	22.80 mg QE/100 g	DPPH: 44.30 FRAP: 1.48	↓ SOD and LPO, ↓ CAT, GPx, GSH in liver ↔ viability on Ishikawa and PC-3 cells and ↑ viability of MCF-7 cells at 0.2–125 μg/mL concentration and incubation for 48 hrs ↑ cytotoxicity on MDAMB 231 cells with a 1 mg/mL dose ↑ cytotoxicity on MCF-7 and SKBR3 cancer cell lines with a 2.5–5 mg/mL dose	[25,108,109,110,166]
Pineapple (*Ananas comosus*)	Malaysia	27.75 mg GAE/100 g	24.74 ± 0.35 mg QE/ 100 g	DPPH: 10.86 (IC_50_ values mg/mL) FRAP (μmol Fe [II]/100 g): 47.92 Total antioxidant capacity (mg AAE/g): 16.12	↓ lipid droplet size between 33.78% and 70.36% ↓ lipid accumulation compared to control in 3T3-L1 murine pre-adipocytes	[176,184]
Rhododendron honey (*Rhododendron* sp.)	Turkey	408.35 mg GAE/100 g		DPPH: 48.95 mg/mL FRAP: 0.0077 mg/100 g honey		[24]
Rosemary honey (*Rosmarinus officinalis* L.)	Spain	102–118 mg GAE/100 g	2.29–5.85 mg CE/100 g	DPPH: 202 μmol TE/100 g FRAP: 215 μmol TE/100 g	↑ apoptosis in HL-60 cells through a ROS-independent cell death pathway ↓ DNA strand breaks (0.1–10 mg mL^−1^, 19–25%) induced by BaP in HepG2 cells	[179,185]
Savory honey (*Satureja montana L.*)	Italy	253.78 mg GAE/100 g	211.68 mg QE/100 g	DPPH, IC_50_ (mg/mL): 10.85 FRAP: (mmol Fe(II)/Kg: 3.702	↔ inhibition activity in BJ (IC_50_ = 27.25–29.70 mg/mL) ↑ inhibition activity in MCF-7 (IC_50_ = 22.85− 44.60 mg/mL), HeLa (IC_50_ = 26.70− 44.20 mg/mL) and in SW620 (25.50–49.70 mg/mL) ↑ apoptosis in SW620 after 48 h with savory honey () ↔apoptosis in MCF-7 and HeLa after 48 h	[186]
Strawberry tree honey *(Arbutus unedo)*	Italy			DPPH: 200.83 (µmol TE/100 g) FRAP: 539.01 µmol TE/100 g TEAC: 392.13 µmol TE/100 g	↓ NF-κB, p-IκBα and Nrf2 expression, ↓ mitochondrial respiration and glycolysis ↑ apoptosis in human colon adenocarcinoma (HCT-116) and metastatic (LoVo) cancer cells ↓ Cell viability ↑ ROS generation ↓ Antioxidant enzyme activity ↓ Nrf2, SOD, catalase, HO-1 ↑ Lipid peroxidation and protein carbonyl content in HCT-116 and LoVo colon cancer cells at 3–12 and 10–40 mg/mL for 48 h ↑ p53, caspase-3, -8, -9, c-PARP1, Bax, Cyto C, FasL ↓ Bcl in HCT-116 and LoVo colon cancer cells at 3–12 and 10–40 mg/mL for 48 h	[187,188]
	Portugal	91.74–117.65	4.09–9.66 mg QE/100 g	DPPH: 40.28–45.20% TEAC:0.39–0.44 mmol TE/100 g ORAC:39.55	n.i.	[100,189]
Sulla honey (*Hedysarum* sp.)	Italy	60.50 mg GAE/100 g	41.88 mg QE/100 g	DPPH, IC_50_ (mg/mL): 54.74 FRAP, (mmol Fe(II)/Kg): 1.299	n.i.	[79,94]
	Italy	11.26 mg GAE/100 g	6.76 mg QE/100 g	DPPH, I%: 66.60	n.i.	[68]
Sunflower honey (Helianthus annus)	Turkey	77.64 mg/100 g GAE	n.i.	DPPH: 19.24 mg/mL FRAP: 0.0047 mg/100 g honey	n.i.	
	Romania	20.0–45.0 mg GAE/100 g	11.53–21.1 mg QE/100 g	DPPH: 60.02–76.95%	↔ inhibition activity on Hep-G2 (32.92%) ↓ inhibition activity onCaco-2 (26.37%) and Hep-G2 (8.47%)	[36,106,171]
	Italy	n.i.	n.i.	DPPH IC_50_: 19.24 mg/mL FRAP: 470 mg/100 g	↓ Visceral fat percentage, hepatoprotective	[24]
	Portugal	36.69 mg GAE/100 g	1.93 mg QE/100 g	TEAC: 3.17 ORAC: 0.41	n.i.	[100]
Thyme honey (*Thymus vulgare*)	Romania, Portugal	18.9–62.91 mg GAE/100 g	17.4–5.62 mg QE/100 g	DPPH IC_50_: 31.4 mg/mL TEAC: 1.59 ORAC: 10.58	↓ viability of Ishikawa and PC-3 cells at 0.2–125 μg/mL concentration and incubation for 48 hrs	[36,100,109]
	Italy	126.55 mg GAE/100 g	73.56 mg QE/100 g	DPPH, IC_50_ (mg/mL): 31.4 FRAP, (mmol Fe(II)/Kg honey): 1.834 ORAC (415–692 μmol of TE/kg)	↓ Tartrate-resistant acid peroxidise activity, hydroxyproline level, oxidative and inflammatory stress	[24,76]
Tree of heaven honey (*Ailanthus altissima*)	Italy	93.72	91.55 mg QE/100 g	DPPH, IC_50_ (mg/mL): 64.09 FRAP, (mmol Fe(II)/Kg honey): 1.268	n.i.	[76]
Tualang honey (*Koompassia excels*)	Malaysia	83.96 mg GAE/ 100 g	50.45 mg QE/ 100 g	DPPH: 9.65 mg AAE/100 g FRAP: 52.39 mg TE/ 100 g	↑ apoptosis (51.2%) at 48 h for MDA-MB-231 cells ↑ apoptosis 55.6% MCF-7 and 56.2%, HeLa cells at 72 h ↓ mitochondrial membrane potential (Δψ_m_) in the cancer cell lines after 24 h of treatment ↑ apoptosis (42.8%) in MCF-7 cells after 24 h ↓ Lipid hydroperoxides, ↓ MDA, ↓ pancreatic SOD, ↑ pancreatic CAT apoptotic cell death in OSCC and HOS cell lines when treated with 2% and 10% honey for 24, 48 and 72 h	[190,191,192,193]
Willow honey (*Salix* *sp.*)	Poland	288.0 mg GAE/kg	n.i.	DPPH, (mmol TEAC/kg): 2.1 FRAP, (mmol Fe(II)/Kg): 0.5	n.i.	[94]
Ziziphus honey (*Ziziphus* sp.)	Egypt, Sudan	81.37−96.99 mg GAE/100 g	5.43−9.15 mg QE/100 g	DPPH: 32.70–86.18% ABTS (IC_50_ = mg/mL): 3.60	↔ inhibitory activity in Caco-2 (34.22%) ↓ inhibitory activity in MCF-7 (29.87%) and Hep-G2 (15.94%)	[168,194]

Note: AAE, ascorbic acid equivalents; TPC, total phenolic content expressed as mg gallic acid/100 g; TFC, total flavonoid content expressed as mg quercetin equivalents/100 g; TRC- mg GAE kg^−1^; TEAC (IC_50_ = mg/mL); ORAC (µmol TE/g); FRAP: lmol FeSO_4_7H_2_O/g; IC_50_, 50% inhibitory concentration; CE; µg of Catechin Equivalent; PMNs, polymorphonuclear neutrophils; ROS, reactive oxygen species; HeLa, cervical carcinoma; MCF-7, breast 696 epithelial adenocarcinoma, metastatic; SW620, colorectal metastatic adenocarcinoma; BJ, normal human 697 skin fibroblasts; OSCC, oral squamous cell carcinomas; HOS, human osteosarcoma;n.i., not identified; ↑ high/increase; ↓ low/decrease; ↔ moderate/medium.

**Table 6:** In vivo studies of different monofloral honey types.

Honey Type	Animal Model or Human Individuals	Mode/Dosage of Honey Administration	Duration	Effects	References
Antioxidant effects					
Tualang honey from Malaysia	Streptozotocin-diabetic Male Sprague-Dawley rats aged 10–12 weeks	Oral administration G1 (Control): Non-diabetic rats received ddH_2_O (0.5 mL). G2 (Diabetic Control): Diabetic rats received ddH2O (0.5 mL). G3: Diabetic (honey: 0.2 g/kg bw). G4: Diabetic (honey: 1.2 g/kg bw). G5: Diabetic (honey: 2.4 g/kg bw).	4 weeks	↓ FPG, ↑ bw gain, ↓ TAS, ↑ activities of CAT, GPx, GR, and GST, ↓ TBARS levels, ↓ thickening of glomerular basement membrane of kidneys	[205]
	Thirty-six male Sprague-Dawley diabetic rats aged 10–12 weeks	G1: Distilled water (0.5 mL) G2: Tualang honey (1.0 g/kg/bw) G3 (Diabetic Control): Distilled water (0.5 mL) G4: (Diabetic) Tualang honey (1.0 g/kg/bw) G5: (Diabetic) Glibenclamide (0.6 mg/kg/bw) + metformin (100 mg/kg/bw) G6: (Diabetic) Glibenclamide (0.6 mg/kg/bw) + metformin (100 mg/kg/bw) + tualang honey (1.0 g/kg/bw)	4 weeks	↓ TBARS levels ↓ superoxide dismutase (SOD) ↓ glutathione peroxidase (GPx) ↑ catalase (CAT) activity ↑ weight gain in G4 and G6 ↓ oxidative stress and damage	[206]
	Forty female Sprague-Dawley rats aged between 45 to 48 days old	rats were given 80 mg/kg DMBA then randomly divided into four groups: G1 (Control): distilled water (vehicle) G2: Tualang honey (0.2 g/kg/bw) G3 Tualang honey (1.0 g/kg/bw) G4: Tualang honey (2.0 g/kg/bw) via oral gavage daily	150 days	↑ apoptotic index in honey-treated groups ↓ concentration of VEGF Protein	[207]
Mad honey (floral source: Rhododendron	sixty Sprague-Dawley female rats were (6–8 months old; 250–300 g)	Group 1 (control): 1 mL of 0.9% NaCl solution was given via intraperitoneal injection Group 2 (GTX): 0.015 mg/kg/bw of Grayanotoxin-III was given via intraperitoneal injection; (*n* = 12) Group 3 (RH1): 0.1 g/kg/bw of RH was given via oral gavage; (*n* = 12) Group 4 (RH2): 0.5 g/kg/bw of RH was given via oral gavage; (*n* = 12) Group 5 (RH3): 2.5 g/kg/bw of RH was given via oral gavage; (*n* = 12)	1 h	↓ SOD, CAT, GTX activity at high dose of honey (2.5 g/kg/bw) ↑ plasma and various tissue MDA levels at high dose of honey	[208]
Pine honey from Turkey	Forty-eight male BALB/c mice, weighing 30–35 g	G1: control G2: 1 g/kg bw/day pine honey G3: 180 mg/kg bw/day (∼1/5LD_50_) trichlorfon G4: 180 mg/kg bw/day trichlorfon plus 1 g/kg bw/day pine honey	21 days	↓ MDA levels, ↑ SOD levels, ↔ CAT and GSH-Px levels in G2 and G4 in liver, kidney, heart and brain tissues	[209]
Malicia honey (*Mimosa quadrivalvis* L)	Thirty-two male Wistar rats at 90 days of age	G1 (control): saline solution via gavage G2 (dyslipidaemic control): saline solution via gavage + dyslipidaemic diet (6% lard, 5% non-hydrolysed vegetable fat, 1% cholesterol and 0.5% cholic acid) G3: honey via gavage G4: honey via gavage + dyslipidaemic diet	5 weeks	↓ Food consumption, ↑ glucose tolerance and SOD activity ↓ TC, LDL and AST levels ↑ beneficial bacteria and organic acids ↓ tissue damage in colon and liver induced by the dyslipidaemic diet	[210]
Fennel honey	Eight female goats s 4–5 months old weighing about 10–24 kg bw	Intravenous administration 70–80 drops/min as rapid infusion of 20% honey solution, daily	4 weeks	↑ GPX, SOD, Lymphocytes (%), Total Leucocytic count (×103/µL), Monocytes (%) ↓ MDA, Plasma globulin (g/dL), Ascorbic acids (mg/dL),	[211]
Chestnut honey	Eighteen Male Wistar rats weighing 150 to 175 g	G1: indomethacin (60 mg/kg, orally) + honey G2: indomethacin + Alimento Supervis G3: indomethacin + Alimento Mieleucalipto G4: indomethacin + sucralfate G5: tap water (5 mL/kg)	7 days	Prevention of indomethacin-induced gastric lesions ↓ ulcer index ↓ microvascular permeability ↓ myeloperoxidase activity of the stomach	[212]
Weight control					
Clover honey	Thirty-six male Sprague-Dawley rats (228.1 ± 12.5 g)	divided by weight into 2 groups (*n* = 18) and provided free access to 1 of 2 diets (20% carbohydrate (by weight of total diet) from either clover honey or sucrose)	33 days	↓ Weight gain and adiposity, ↓ TG ↑ non-HDL-C levels	[213]
Acacia and Gelam honey	Seven-week-old male Sprague-Dawley rats, with body weight ranging from 200 to 220 g	G1: normal control G2: high fat diet G3: high fat diet rats fed with Gelam honey, G4: high fat diet rats fed with Acacia honey, G5: high fat diet rats treated with orlistat	4 weeks	↓ in excess weight gain and adiposity index ↓ plasma glucose, triglycerides, and cholesterol, plasma leptin and resistin, liver enzymes, renal function test, and relative organ weight	[214]
Honeydew honey	Fifty-five Sprague Dawley rats, aged approximately 8 weeks	G1: sugar-free diet G2: 7.9% sucrose G3: 10% honey	365 days	Similar weight gain and body fat in honey and control group; ↓ HbA1c, ↑ HDL-C	[215]
Tualang honey from Malaysia	male Wistar albino rats (*n* = 40) weighing 160–180 g	G1 (control): standard laboratory diet and drinking water ad libitum G2 (Tualang Honey): Orally administered (3 g/kg) for 45 days. G3 (ISO): Animals were subcutaneously injected with ISO (85 mg/kg) on the 44th and 45th days (at an interval of 24 h) G4 (TH + ISO): Animals were orally treated with TH (3 g/kg) for a period of 45 days followed by subcutaneous injection of ISO (85 mg/kg) on the 44th and 45th days (at an interval of 24 h)	2 days	↑ Antioxidant enzyme levels in heart tissue ↓ LPO	[216]
Pineapple	Forty-eight healthy Sprague Dawley male rats weighting 280–220 g	G1 (control): water *ad libitum* G2: 2000 mg/kg bw pineapple honey for 24 h G3: 2000 mg/kg bw adulterated honey A G4: 2000 mg/kg bw adulterated honey B	14 days	↓ cholesterol levels (18.94 ± 3.6 mmol/L) ↓ triglycerides (13.5 ± 1.5 mmol/L) and glucose (8.0 ± 1.5 mmol/L) levels Early mortality in G3 and G4 of five rats	[217]
Hypercholesterolemia and anti-diabetic					
Mad honey (floral source: *Rhododendron ponticum*) from Turkey	Streptozotocin-diabetic rats and non-diabetic rats	Honey given 50 mg/ kg/day (2 mL mad honey dissolved in distilled water)	3 days	Significant ↓Glucose in both diabetic and non-diabetic rats	[218]
Clover and Citrus honey from Egypt, and Ziziphus honey from Yemen and Pakistan	Type 2 diabetics human subjects (*n* = 38)	Solely treated with honey 2 g/kg/day, orally, before meals twice daily, no antidiabetic medicines were running	Between 0.42–13.5 years	↑ Glucose, ↔ RBG, ↔ TG, ↔ TC, ↔ HDL, ↔ LDL, ↔ TC/HDL and LDL/HDL ratios, ↓ SBP, ↓ DBP, ↓ bw, prevented ketoacidosis, hyperglycaemic hyperosmolar state, and macrovascular complications (particularly coronary heart disease)	[219]
Kanuka honey	Type 2 diabetes human subject, weight and blood samples	G1: 53.5 g (three tablespoons) of kanuka honey G2: mixture of a formulated honey (53.5 g) comprised of 4.5 g food grade cinnamon, 200 µg chromium polynicotinate and 120 mg magnesium citrate mixed with 100% kanuka honey	40 days	↓ weight ↓ blood pressure ↓ TC Improve blood lipid profile	[220]
Clover honey from Egypt	Type 1 diabetics human subjects (*n* = 20) aged 4–18 years	Orally administered honey (0.5 mL/kg bw/day)	12 weeks	↓ FSG, ↓ Glycosylated hemoglobin, ↓ SSFT, ↓ TC, ↓ LDL, ↑ FCP, ↑ PCP	[221]
	Single case report of a patient with CHD, hypertension and type 2 diabetes	Given 150 g honey daily, orally	11 years	Controlled RBG, blood pressure, improved/stabilized CHD, prevented ketoacidosis or hyperosmolar coma.	[222]
Coriander honey from Egypt	210 male Swiss albino mice weighting 22–25 g with serial intraperitoneal passage Ehrlich ascites carcinoma cells	G1 (normal control): orally dose of 50 µl/ mouse normal saline daily G2 (coriander control): daily dose of 500 mg/kg/mouse through oral administration G3 (5-FU control): daily dose of 20 mg/kg/mouse of 5-flurouracil as standard anticancer G4 (EAC control): was inoculated intraperitoneally with a single dose of EAC cell line (2 × 106 cells/mouse) + normal saline G5: Coriander + EAC G6: 5-FU + EAC	21 days	↑ IgM, IgG and IgA levels ↑ Phagocytic activity ↑ skin thickness	[223]
Tualang honey from Malaysia	Ten female Sprague-Dawley rats (age 6–8 weeks) weighing 140–170 g	G1 (HCD): 12% cholesterol diet G2 (HCD+TH): 12% cholesterol diet along with oral daily dose of 1.4 g/kg/day of tualang honey by gavage	6 weeks	↓ TC and TG compared to the control at 7 day ↓ Serum creatinine level copared to G1 after 48 h No structural effect in the HCD-fed rats	[224]
Buckwheat honey	Male Kunming mice (18–22 g)	G1 (Control): distilled water via gavage at 0.22 mL/10 g bw G2 (CCl4-treated mice): distilled water via gavage at 0.22 mL/10 g bw G3: 0.22 g/10 g bw of buckwheat honey G4: 0.5 mg/10 g bw of silymarin via gavage	10 weeks	serum lipoprotein oxidation inhibition aspartate aminotransferase and alanine aminotransferase activities inhibition ↑ serum oxygen radical absorbance capacity ↓ Hepatic malondialdehyde ↑ GSH-Px and SOD activities lymphocyte DNA damage induced by carbon tetrachloride inhibition	[225]
Coriander honey	21 patients between 20 and 85 years	G1 (*n* = 22): probiotics G2 (*n* = 21): probiotic combination with honey G3 (*n* = 24): placebo	6 weeks	↓ RBC, WBC, platelet levels ↓ Iga levels from 236.3 (58.6) to 206.3 (64.3) mg/dL	[226]
Antiproliferative					
Manuka honey	male mice at 8–12 weeks of age	50% (*w*/*v*) manuka honey intravenously, 10 mg/kg taxol twice weekly	3–4 weeks	↑ Caspase-3 ↑ Survival rate	[185]

DMBA, carcinogen 7,12-dimethylbenz(α)anthracene; VEGF, vascular endothelial growth factor; HDL-C, high density lipoprotein cholesterol; HFD, high-fat diet; FPG, fasting plasma glucose; bw, body weight; TAS, total antioxidant status; CAT, catalase; GPx, glutathione peroxidise; GR, glutathione reductase, GST glutathione-*S*-transferase; SOD, superoxide dismutase; LPO, Lipid peroxidation; ISO,Isoproterenol; HbA1c, Haemoglobin A1c; HDL-C, high density lipoprotein cholesterol; LDL-C, low density lipoprotein cholesterol; VLDL-C, very low density lipoprotein cholesterol; TC, total cholesterol; TG, triglycerides; LPO, lipoprotein oxidation; HCD, high cholesterol diet; AST, aspartate aminotransferase; CRP, C-reactive protein; SSFT, subscapular skin fold thickness; FSG, fasting serum glucose; FCP, fasting C-peptide; PCP, 2-h postprandial C-peptide; LDL, low density lipoprotein cholesterol; CHD, Coronary Heart Disease; RBG, random blood glucose; HDL, high density lipoproteins; SBP, systolic blood pressure; DBP, Diastolic Blood Pressure; ↑ high/increase; ↓ low/decrease; ↔ moderate/medium.
